# Mobile charges in MoS_2_/high-k oxide transistors: from abnormal instabilities to transient negative differential resistance

**DOI:** 10.1038/s44172-026-00716-2

**Published:** 2026-07-02

**Authors:** Shaokai Zhou, Haihui Cai, Yehao Wu, Yufeng Min, Renchen Yuan, Yezhu Lv, Jianming Huang, Yuanyuan Shi, Yury Yuryevich Illarionov

**Affiliations:** 1 Laboratory of 2D Optoelectronics and Nanoelectronics (L2DON), State Key Laboratory of Quantum Functional Materials, Department of Materials Science and Engineering, Southern University of Science and Technology, Shenzhen, China; 2 School of Integrated Circuits, Anhui Province Key Laboratory of Integrated Circuit Science and Technology, University of Science and Technology of China, Hefei, China

**Keywords:** Electronic devices, Electronic devices

## Abstract

Molybdenum disulfide (MoS_2_) field-effect transistors with high-*k* oxides lag behind silicon standards in stability due to traps causing clockwise hysteresis. While suppressing this effect is mandatory for logic devices, here we show an alternative strategy where initial hysteresis is dynamically overcome by stronger counterclockwise hysteresis coupled with memory-like transient negative differential resistance. We compare back-gated transistors using HfO_2_ and Al_2_O_3_ gate insulators up to 275 °C. At 175 °C, devices with HfO_2_ exhibit dominant counterclockwise dynamics and transient negative differential resistance. Our compact model suggests this behavior is caused by the drift of mobile oxygen vacancies within HfO_2_. This mechanism overrides initial hysteresis, revealing a pathway to memory-like functionality enhanced by narrower voltage sweep ranges. In contrast, transistors gated with Al_2_O_3_ display only minor counterclockwise dynamics even at 275 °C due to higher vacancy migration barriers, maintaining superior stability. Our results reveal an insulator selection paradigm: Al_2_O_3_ is better suited for logic devices, whereas HfO_2_ counterparts can serve as active layers for memory components.

## Abstract (web summary)

Shaokai Zhou and colleagues compare hysteresis in MoS_2_ transistors with HfO_2_ and Al_2_O_3_ gate insulators up to 275 °C. HfO_2_ devices exhibit memory-like transient negative differential resistance, while Al_2_O_3_ devices remain stable, establishing a choice for logic and memory applications.

## Introduction

Moore’s scaling of silicon-based field-effect transistors (FETs) is approaching its fundamental limits due to sizable short-channel effects and mobility degradation in thin Si nanosheets^1^. This creates an urgent need for alternative channel materials, such as 2D semiconductors^2,3^, which could replace or substitute Si in next-generation integrated circuits^2,4,5^. Atomically thin MoS_2_ that enables superior electrostatic control and offers reasonable compatibility with conventional high-k oxides, such as HfO_2_ and Al_2_O_3_, has emerged as a leading candidate for 2D n-FETs^3,6,7^. Furthermore, recent research advances^2,8–11^ have made it possible to move forward from lab prototypes to the trial integration of the MoS_2_ FETs with HfO_2_ and Al_2_O_3_ into the industrial process lines^6,7,12–14^. However, these devices still face severe stability and reliability limitations caused by various defects in gate insulators, with border oxide traps^15,16^ and mobile charges^17,18^ being the most ubiquitous. As a result, the pathway of MoS_2_ FETs and other 2D devices to mass production is delayed since it is still hard to compete with Si technologies in stable long-term operation.

A powerful diagnostic tool to benchmark stable operation of MoS_2_ FETs can be offered by a comprehensive analysis of time-dependent hysteresis dynamics in the gate transfer (*I*_D_–*V*_G_) characteristics^16,17,19,20^. Hysteresis serves as a sensitive indicator for the stability of MoS_2_ FETs and can also reveal valuable physical phenomena that could be exploited for beyond-FET applications. That is because it directly reflects threshold voltage fluctuations and captures key information about dynamic processes such as charge trapping by oxide defects and drift of mobile charges^18^. However, many existing studies on 2D FETs miss this opportunity to get in-depth information and use just a single sweep at a random sweep rate, which only allows to speculate that hysteresis is “negligible” or “near-zero”^21–26^ with no relation to the real physical picture. As a result, no systematic comparison of time- and temperature-dependent hysteresis dynamics in MoS_2_ FETs with HfO_2_ and Al_2_O_3_ insulators under identical conditions can be found in the literature. This prevents a clear understanding of intrinsic stability concerns introduced by these two most widely used high-k gate oxides and thus impedes the formulation of targeted optimization strategies that could finally enable MoS_2_/high-k FETs with competitive stability. Furthermore, the lack of detailed studies focused specifically on abnormal counterclockwise (CCW) dynamics impedes possible ways for the practical use of this phenomena in memory devices as an alternative to suppression.

Here, we perform a systematic comparison of hysteresis dynamics in MoS_2_/HfO_2_ and MoS_2_/Al_2_O_3_ FETs fabricated using identical methods at sweep times up to tens of kiloseconds and temperatures varied from 25 to 275 °C, and support our findings with bias stress measurements. Our results reveal that at room temperature, both devices exhibit a clockwise (CW) hysteresis that is caused by charge trapping and thus defined by energetic alignment of oxide defect bands. However, at 175 °C, our MoS_2_/HfO_2_ FETs exhibit a change of hysteresis to the CCW direction, coming together with transient negative differential resistance (TNDR) and self-doping effects. Our qualitative compact model for mobile charges in oxides nicely reproduces the CCW hysteresis with all related phenomena and suggests the drift of positive oxygen vacancies in HfO_2_ as the primary reason that may also result in controllable memory-like TNDR dynamics. However, in Al_2_O_3_ insulators, similar trends are barely visible even at temperatures as high as 275 °C due to larger migration barriers of the same vacancies, thus making this insulator more suitable to mitigate detrimental negative shifts of *V*_th_ under the positive bias stress.

## Results and discussion

### Investigated devices and measurement techniques

**Fig. 1:**
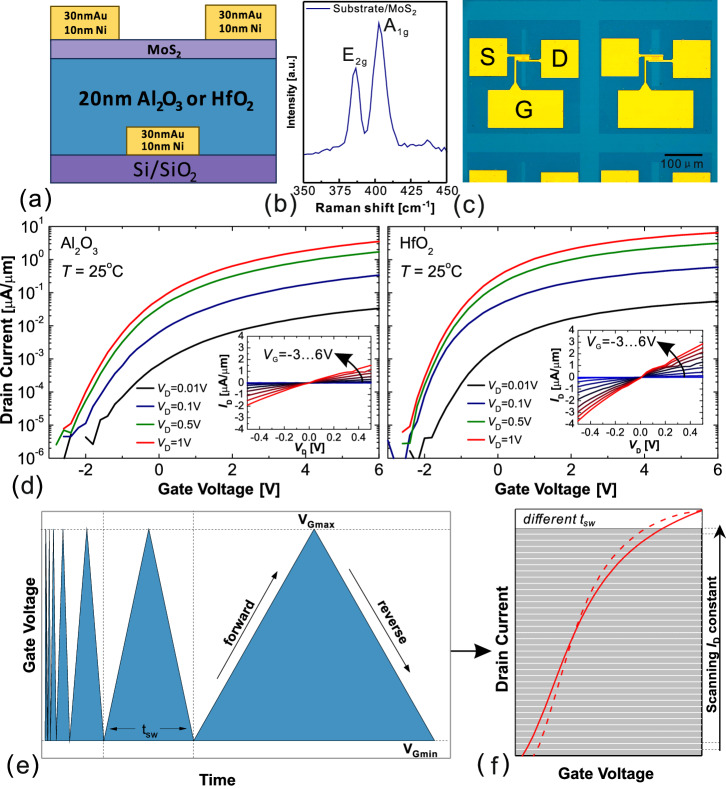
Device structure, basic characteristics, and hysteresis measurement technique. **a** Schematic layout of our back-gated MoS_2_/HfO_2_ and MoS_2_/Al_2_O_3_ FETs fabricated using the same process. The channel is made of MoS_2_ films grown by chemical-vapor deposition (CVD) and taken from the same batch. **b** Raman spectrum that confirms single-layer thickness of the MoS_2_ channel. **c** Optical image of four devices inside the array containing tens of field-effect transistors (FETs). **d** *I*_D_–*V*_G_ characteristics of MoS_2_ FETs with Al_2_O_3_ (left) and HfO_2_ (right) measured at different *V*_D_. The insets show the corresponding *I*_D_–*V*_D_ curves. **e** Schematics of the subsequent *V*_G_ sweeps with increased *t*_sw_ used in our hysteresis measurements. In some measurements, the maximum *t*_sw_ that we have reached was above 10 ks. **f** Schematics of our universal mapping method^27^, which suggests scanning the *I*_D_ values to obtain minimum (lower universal hysteresis function (UHF)) and maximum (upper UHF) from the extracted family of Δ*V*_H_(1/*t*_sw_) curves.

We fabricated back-gated MoS_2_ FETs with HfO_2_ and Al_2_O_3_ gate insulators with the schematic layout shown in Fig. 1a. To enable consistent comparison of the device stability, for both types of FETs, we used commercial single-layer MoS_2_ films, as confirmed by Raman measurements (Fig. 1b), grown by chemical-vapor deposition (CVD), taken from the same batch, and also employed the identical fabrication processes. First, 10 nm Ni/30 nm Au local back gate electrodes were deposited on a pretreated Si/SiO_2_ substrate via photolithography and e-beam evaporation. Then a 20 nm-thick HfO_2_ or Al_2_O_3_ insulator was grown by atomic layer deposition (ALD) at 250 °C. Next, the MoS_2_ film was transferred through a wet transfer process, followed by residual removal and patterning of the channel via photolithography and reactive ion etching. Finally, the source/drain electrodes were patterned by photolithography, e-beam evaporation and lift-off process. The obtained arrays with hundreds of MoS_2_ FETs of both types (Fig. 1c) with channel length *L* and width *W* varied between 5 and 100 μm allow us to perform systematic measurements while verifying the reproducibility of all observed trends.

The electrical characterization of our MoS_2_ FETs was performed in vacuum (~5 × 10^−6^ torr) and in complete darkness. First, we have checked the *I*_D_–*V*_G_ characteristics at different drain voltages, *V*_D_. As shown in Fig. 1d, for both MoS_2_/Al_2_O_3_ and MoS_2_/HfO_2_ FETs, decent performance with on/off current ratios of about 10^6^ is obtained, while the output (*I*_D_–*V*_D_) curves also look reasonable. Next, we measured the hysteresis using subsequent double sweeps of the *I*_D_–*V*_G_ characteristics at *V*_D_ = 0.1 V with sweep times *t*_sw_ increased from tens of seconds to over 10 ks (Fig. 1e), while repeating the measurements with different *V*_G_ sweep ranges. The hysteresis width Δ*V*_H_ is defined as the gate voltage difference between the forward and reverse *I*_D_–*V*_G_ sweeps at a fixed drain current. For consistent extraction of the hysteresis width Δ*V*_H_ vs. 1/*t*_sw_ dependences, we used our standardized universal mapping method^27^. This approach suggests scanning the constant current value to extract the lower and upper universal hysteresis functions (UHFs)^27^, as schematically shown in Fig. 1f (see more details in “Methods”). In contrast to the conventional constant current approach used in our previous studies^17,28^, the mapping method captures complex hysteresis dynamics like the CW/CCW switching caused by two competing mechanisms, such as charge trapping and drift of mobile charges observed in this work.

### Hysteresis dynamics at room temperature

**Fig. 2:**
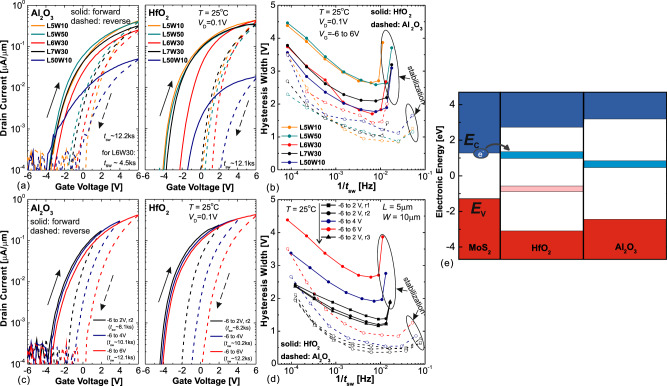
Room-temperature hysteresis dynamics. **a** Double sweep *I*_D_–*V*_G_ characteristics of 5 MoS_2_ field-effect transistors (FETs) with Al_2_O_3_ (left) and HfO_2_ (right) with different *L* and *W* measured using the slowest achieved sweeps at 25 °C. A purely clockwise (CW) hysteresis is present for all devices. **b** The corresponding Δ*V*_H_ vs. 1/*t*_sw_ dependences showing that while there is some device-to-device variability for both types of devices, overall the CW hysteresis is smaller for the devices with Al_2_O_3_. **c** Double sweep *I*_D_–*V*_G_ characteristics of the representative MoS_2_ FETs with Al_2_O_3_ (left) and HfO_2_ (right) measured using the slowest *t*_sw_ and different *V*_G_ sweep ranges. **d** The corresponding Δ*V*_H_ vs. 1/*t*_sw_ curves confirm that for the MoS_2_/Al_2_O_3_ FETs, hysteresis is smaller for all *V*_G_ sweep ranges. Remarkably, the results for −6 to 2 V sweep range repeated in three rounds (r1, r2, and r3) in the beginning and in the end of the experiment (as illustrated by the arrow next to the legend) show perfect reproducibility. **e** Band diagram showing relative energetic alignments between the conduction band edge *E*_C_ of the MoS_2_ n-channel and known fundamental defect bands in HfO_2_ and Al_2_O_3_^16,29^. The upper defect band of HfO_2_ is closer to the *E*_C_ of MoS_2_, while the defect band of Al_2_O_3_ is situated deeper. This explains the smaller CW hysteresis in the latter case, since deeper traps in Al_2_O_3_ get activated at slower sweeps.

First, we reveal the impact of gate insulator and device-to-device variability on the hysteresis dynamics at room temperature by examining MoS_2_ FETs with different *L* and *W*. In Fig. 2a, we show the double sweep *I*_D_–*V*_G_ characteristics measured for 5 MoS_2_/Al_2_O_3_ and 5 MoS_2_/HfO_2_ devices using the slowest achieved *t*_sw_ and the *V*_G_ sweep range of −6 to 6 V. A purely CW hysteresis is observed in all cases. Then we use our full measurement datasets consisting of 8 sweeps with *t*_sw_ ranging between tens of seconds and over ten kiloseconds to perform the full mapping of hysteresis dynamics. As shown in Fig. S1 in the Supplementary Information (SI), for both types of our devices, only CW hysteresis is present within the whole range of *t*_sw_, and thus the Δ*V*_H_(1/*t*_sw_) dependences can be consistently expressed with the upper UHFs. In Fig. 2b, we show the resulting Δ*V*_H_(1/*t*_sw_) curves for all 10 devices. While some variability is present within 5 devices of each type, there is no obvious correlation between the hysteresis magnitude and the channel dimensions. Furthermore, from Fig. 2b, we can reliably conclude that the CW hysteresis in MoS_2_ FETs with Al_2_O_3_ is generally smaller than in their counterparts with HfO_2_. To get more insights into this difference, we next select representative MoS_2_ FETs of both types and measure the hysteresis using different *V*_G_ sweep ranges. The double sweep *I*_D_–*V*_G_ characteristics measured at the slowest *t*_sw_ and the corresponding Δ*V*_H_(1/*t*_sw_) dependences are shown in Fig. 2c, d, respectively. We can see that for both types of our MoS_2_ FETs, the CW hysteresis becomes larger for wider *V*_G_ sweep ranges and also increases for slower sweeps that is the typical feature of charge trapping by oxide defects^16^. At the same time, it is again reconfirmed that for the MoS_2_/Al_2_O_3_ FETs, the CW hysteresis is generally smaller and starts to increase at considerably lower sweep frequencies for all three sweep ranges used. Remarkably, the results for the narrowest *V*_G_ sweep range of −6 to 2 V measured before and after the use of wider *V*_G_ sweep ranges are nicely reproducible. However, stabilizing behavior of the devices, which appears as a larger hysteresis measured for the first sweeps of each round, has to be noted in all cases.

The observed difference in room temperature hysteresis dynamics between MoS_2_ FETs with Al_2_O_3_ and HfO_2_ gate insulators goes in line with the energetic alignment of oxide defect bands with respect to the conduction band edge *E*_C_ of MoS_2_, as shown in Fig. 2e. These defect bands have fundamental positions inside the bandgap of high-k oxides, which are relatively well known but may slightly depend on the oxide quality and growth conditions^16,29^. Namely, the upper defect band of HfO_2_ is close to the *E*_C_ of MoS_2_. This facilitates trapping of electrons from the MoS_2_ channel by HfO_2_ defects^30^, which explains a sizable CW hysteresis observed in our MoS_2_/HfO_2_ FETs already at faster sweeps. In contrast, the defect band of Al_2_O_3_ is over 0.5 eV below the *E*_C_ of MoS_2_, which drastically increases the energy barrier for charge trapping by oxide traps. That is why the CW hysteresis in our MoS_2_/Al_2_O_3_ FETs is generally smaller as compared to their counterparts with HfO_2_. Furthermore, Δ*V*_H_ starts increasing at smaller sweep frequencies as these deeper traps in Al_2_O_3_ have larger capture/emission time constants and thus need more time to get activated^17,27^.

### MoS_2_/HfO_2_ FETs at high temperatures: memory-like performance

#### Experimental classification of the CCW hysteresis dynamics

**Fig. 3:**
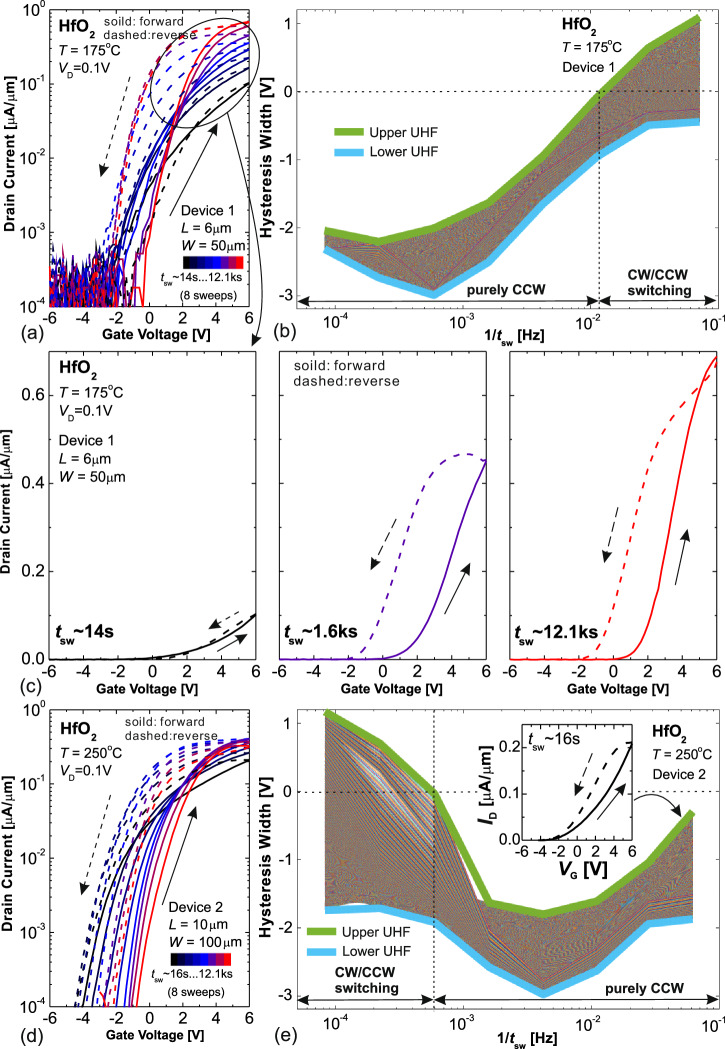
High-temperature hysteresis dynamics. **a** Double sweep *I*_D_–*V*_G_ characteristics of our MoS_2_/HfO_2_ field-effect transistor (FET) measured at *T* = 175 °C using 8 subsequent sweeps with *t*_sw_ up to 12.1 ks. The hysteresis dynamics change from the clockwise/counterclockwise (CW/CCW) switching at faster sweeps to the purely CCW hysteresis at slower sweeps. **b** The corresponding mapping results, which clearly reveal the frequency ranges of both hysteresis dynamics and a distinct maximum of the CCW hysteresis. **c** Linear scale *I*_D_–*V*_G_ curves corresponding to (**a**), which show development of the CCW hysteresis, i.e., a weak effect at fast *t*_sw_, a transient negative differential resistance (TNDR) behavior at moderate *t*_sw_, and an increase of *I*_ON_ with localization and decay of the CCW hysteresis at slow *t*_sw_. **d**, **e** The related results measured at *T* = 250 °C C. The CCW hysteresis maximum is shifted to faster sweeps, and thus the CW/CCW switching is observed at slower sweeps. The inset in (**e**) highlights that TNDR is not observed within the *t*_sw_ range used since mobile charges are too fast.

In Fig. 3a, we show the double sweep *I*_D_–*V*_G_ characteristics of our MoS_2_/HfO_2_ FET measured at *T* = 175 °C using 8 subsequent sweeps with *t*_sw_ up to 12.1 ks. The corresponding hysteresis mapping results provided in Fig. 3b reveal a totally different hysteresis behavior as compared to room temperature. Namely, the hysteresis changes from the CW/CCW switching at fast sweeps to the purely CCW one at slow sweeps. This is followed by a sizable increase in the ON state current from one sweep to another (Fig. 3a), which suggests progressive n-type doping of MoS_2_ during the measurements. We note that this current increase is unlikely to be caused by self-heating effects, since for *V*_D_ = 0.1 V and *I*_D_ up to tens of *μA* the Joule heating power would be only several *μW*. Remarkably, our full mapping results clearly separate the frequency regions in which the CW/CCW switching and purely CCW hysteresis are present, as in the former case, the upper and lower UHFs are of opposite signs, and in the latter case, they are both negative. At the same time, a distinct maximum of the CCW hysteresis is visible. This is, in part, similar to our previous observations for MoS_2_/SiO_2_ FETs^17^ and hints at the contribution coming from mobile charges in the oxide. Furthermore, in Fig. 3c, we show the representative *I*_D_–*V*_G_ characteristics in a linear scale and reveal that for the moderate *t*_sw_ an TNDR effect is present during the reverse sweep. As will be discussed below, this memory-like behavior appears if positive mobile charges approaching the channel side of HfO_2_ lag behind *V*_G_ changes near ${V}_{{{\rm{Gmax}}}}$. However, for slower *t*_sw_ a stronger increase of the ON current with no TNDR effect is observed because the self-doping peaks at less positive *V*_G_ during the forward sweep, and thus by reaching ${V}_{{{\rm{Gmax}}}}$ the charges are already trapped at the channel side of oxide. The related results obtained at *T* = 250 °C (Fig. 3d, e) show that the maximum of CCW hysteresis is shifted towards faster sweeps because of thermal activation of mobile charges. As a result, the CCW contribution becomes smaller at slow sweeps, which results in the CW/CCW switching since the charge trapping contribution is still present. However, as the mobile charges are too fast at this temperature, we do not observe any sizable TNDR effect. For instance, the inset of Fig. 3e suggests that only some signs of the current plateau are visible at the beginning of the reverse sweep, even at the fastest *t*_sw_ used.

We also note that the CCW hysteresis dynamics starts to appear in most of our MoS_2_/HfO_2_ FETs at *T* = 125 °C  for slow *t*_sw_, with some outlying devices with likely more vacancies in the oxide showing TNDR behavior already at this temperature. This potentially suggests that by varying the density of oxygen vacancies via adjusting the ALD growth conditions, one can tune the TNDR performance. However, *T* = 175 °C appears to be the optimum for detailed analysis as the CCW hysteresis becomes dominant at this temperature. Furthermore, the observed CCW dynamics are well reproducible between different devices, which simplifies analysis if certain devices get failed during the measurements. More detailed results on temperature dependence and reproducibility between different devices can be found in Figs. S2–S3 in the SI.

#### Understanding the observed trends via compact modeling

To reveal the physical origin of the complex high-temperature hysteresis dynamics in our MoS_2_/HfO_2_ FETs, we implement a compact model for the drift of mobile ions in the gate oxide while disregarding the CW contribution coming from oxide traps that is studied elsewhere by considering oxide defect bands^17,30^. The model incorporates thermally activated hopping of mobile charges with the diffusion coefficient given as

1 $$D={D}_{0}\exp \left(-\frac{q{E}_{{{\rm{A}}}}}{{k}_{{{\rm{B}}}}T}\right)$$

where *D*_0_ is a pre-factor coefficient and *E*_A_ is the energy activation barrier for the migration of ions. Following the Einstein relation, this diffusion coefficient sets the mobility of ions and consequently their drift velocity under the electric field given by the applied *V*_G_. As a result, the position of mobile charges in the oxide *x*(*t*) can be calculated at any time point and used to elaborate the resulting time-dependent threshold voltage shift as

2 $$\Delta {V}_{{{\rm{th}}}}(t)=-\frac{{Q}_{\max }}{{C}_{{{\rm{ox}}}}}\left(1-\frac{x(t)}{{d}_{{{\rm{ox}}}}}\right)$$

where ${Q}_{\max }=q{N}_{{{\rm{mob}}}}{d}_{{{\rm{ox}}}}$, *N*_mob_ is the concentration of mobile charges in the oxide, *d*_ox_ and *C*_ox_ are oxide thickness and capacitance, respectively. The minus sign takes into account that we consider positive charges, which should create a negative Δ*V*_th_ that will have a maximum when all ions accumulate near the channel/oxide interface (i.e., *x* = 0). Finally, this ion-induced Δ*V*_th_ is used for piecewise calculation of the drain current as

3 $${I}_{{{\rm{D}}}}({V}_{{{\rm{G}}}},t)=\left\{\begin{array}{ll}{I}_{\min }\cdot \exp \left(\frac{{V}_{{{\rm{G}}},{{\rm{eff}}}}-{V}_{{{\rm{th}}}}-\Delta {V}_{{{\rm{th}}}}}{SS}\right)\quad &{V}_{{{\rm{G}}},{{\rm{eff}}}}\le {V}_{{{\rm{th}}}}+\Delta {V}_{{{\rm{th}}}}\\ {\mu }_{{{\rm{eff}}}}\cdot {n}_{2{{\rm{D}}}}\cdot q\cdot \frac{W}{L}\cdot {V}_{{{\rm{D}}}}\hfill \quad &{V}_{{{\rm{G}}},{{\rm{eff}}}} > {V}_{{{\rm{th}}}}+\Delta {V}_{{{\rm{th}}}}\end{array}\right.$$

where the carrier density *n*_2D_ = *C*_eff_(*V*_G,eff_ − *V*_th_ − Δ*V*_th_)/*q* with effective capacitance *C*_eff_ accounting for the quantum capacitance effects in the MoS_2_ channel and the capacitance of interface states. The effective gate voltage *V*_G,eff_ = *V*_G_ − *Q*_it_/*C*_ox_ is used to consider possible screening by interface charges. For simplicity, the subthreshold swing SS, minimum current ${I}_{\min }$, and carrier mobility *μ*_eff_ were inset as input parameters to the model, and *V*_th_ was considered to be 0.5 V above the calculated flatband voltage *V*_FB_. The use of this approach allowed us to simulate the double sweep *I*_D_–*V*_G_ characteristics while considering different *t*_sw_. The complete description of our compact model with all parameters can be found in the SI.

We suggest that the observed CCW hysteresis, TNDR, and negative *V*_th_ shifts in our MoS_2_/HfO_2_ devices originate exclusively from mobile charge migration within the high-k oxide, being independent of the MoS_2_ channel. Remarkably, the impact of mobile contaminants in gate oxides on the device stability has been known from early Si FETs^31^. Our results are most consistent with field-driven drift of positively charged oxygen vacancies (${V}_{O}^{+}$, ${V}_{O}^{2+}$) within the HfO_2_ gate dielectric, as they are widely present in ALD-grown high-k oxides^32^. Furthermore, these defects have already been shown to cause the CCW hysteresis dynamics in MoS_2_ FETs with crystalline SrTiO_3_ insulators^33^, and in amorphous oxides, their activation energies are expected to be lower. Alternative mobile species that would also cause the CCW hysteresis could include mobile protons (*H*^+^) and alkali ions, such as *K*^+^^17,20^. However, *H*^+^ is typically present in thin film FETs, being much faster and causing the CCW hysteresis at room temperature^34^. As for *K*^+^ ions, which also cause CCW hysteresis at elevated temperatures^17^, they may penetrate to gate insulators during direct CVD growth of the MoS_2_ channels with potassium salts used as catalysts, which is not the case of our work that uses transferred MoS_2_ films. Furthermore, in the previous works^17,20^, no TNDR effects have been reported but rather a simple CCW hysteresis, likely because *K*^+^ contaminants have smaller concentrations as compared to oxygen vacancies.

**Fig. 4:**
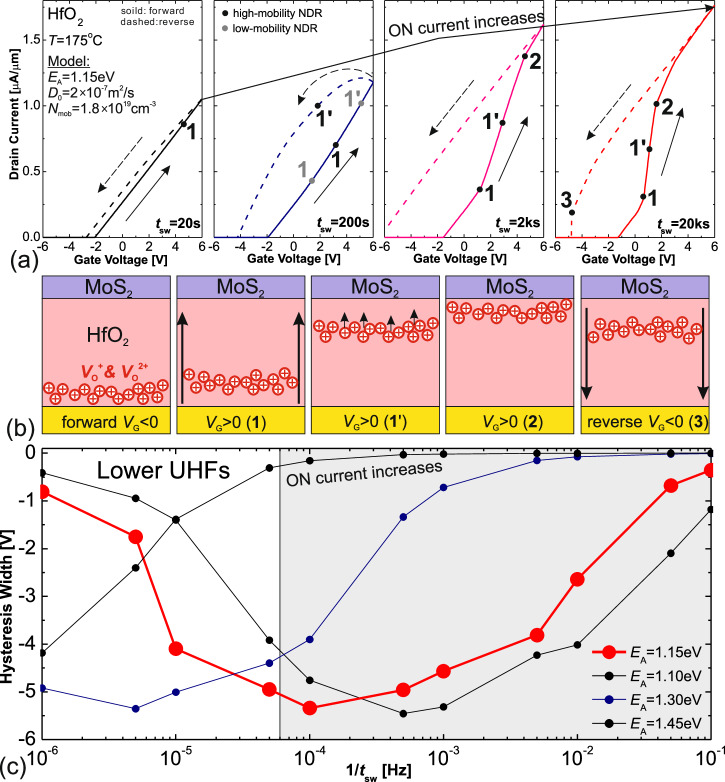
Compact model interpretation. **a** Double sweep *I*_D_–*V*_G_ characteristics of the MoS_2_/HfO_2_ field-effect transistor (FET) simulated with our compact model using different *t*_sw_ and *T* = 175 °C. The trends are qualitatively similar to our experimental results shown in Fig. 3c. **b** Schematics that illustrate the drift of positive oxygen vacancies in the oxide. At negative the *V*_G_, they are concentrated at the gate side of HfO_2_. Then, when *V*_G_ becomes positive, bulk motion towards the MoS_2_ channel starts if *t*_sw_ is slow enough (state 1). However, when approaching MoS_2_, the charges start feeling the interface effects and thus slowing down (state 1′), before finally getting trapped at the channel side (state 2). The dynamics of the counterclockwise (CCW) hysteresis and its side features, such as the transient negative differential resistance (TNDR) effect and *I*_ON_ increase, are determined by the relative positions of the points 1, 1′, and 2 on the *I*_D_–*V*_G_ curves that depend on *t*_sw_. Also, if the sweep is too slow, the drift of mobile charges back to the gate may start (state 3) when *V*_G_ becomes negative during the reverse sweep. **c** The lower universal hysteresis functions (UHFs) extracted by the mapping method from the series of *I*_D_–*V*_G_ characteristics simulated with different *E*_A_. While a maximum of the CCW hysteresis, similar to Fig. 3b, is revealed, a larger *E*_A_ shifts it to the slower sweep frequencies and vice versa. The gray box marks the typical measurement range.

We have identified that the results of Fig. 3 can be qualitatively reproduced with the model by using *E*_A_ = 1.15 eV, which is close to the literature values for oxygen vacancies in HfO_2_^35^, and also physically feasible *N*_mob_ = 3 × 10^19^ cm^−3^ and *D*_0_ = 2 × 10^−7^ m^2^/s. Note that for *K*^+^ ions in SiO_2_ in our previous work^17^ we used *N*_mob_ = 10^18^ cm^−3^ as these are process-induced contaminants present in smaller amounts which are indeed insufficient to cause TNDR (see Fig. S4 in the SI), unlike native HfO_2_ defects, such as ${V}_{O}^{+}$ and ${V}_{O}^{2+}$. The simulated double sweep *I*_D_–*V*_G_ characteristics, which exhibit TNDR behavior and the ON current increase, are shown in Fig. 4a. These results could be interpreted based on the schematics provided in Fig. 4b. Namely, at negative *V*_G_, positive mobile charges are concentrated at the back gate side of HfO_2_. As *V*_G_ becomes positive, they start moving closer to the MoS_2_ channel (state 1). Then, if *t*_sw_ is too short for the ions to respond, they are far from the channel, and thus the CCW hysteresis is small. However, for slower *t*_sw_, the CCW dynamics further develop, as the ions approach the channel, making the CCW hysteresis larger, and at the same time start slowing down due to the interface effects (state 1′). In our compact model, this is considered by reducing the rate of mobile charges near interfaces (see Eq 5 in the SI). Then, if ${V}_{{{\rm{Gmax}}}}$ is within the free bulk motion of ions, i.e., between the states 1 and 1′, high-mobility TNDR memory effects appear. This is because ions continue moving towards the channel fast when the sweep direction changes. As a result, *I*_D_ increases against the *V*_G_ change as the cumulative Δ*V*_th_ given by Eq. 2 becomes more negative. Then the precondition for TNDR is that mobile charges movement lags behind the *V*_G_ change. This can also be realized if the state 1′ is just before ${V}_{{{\rm{Gmax}}}}$ as the ions are still moving, though much slower, i.e., low-mobility TNDR. However, if *t*_sw_ is increased further, mobile charges finally reach the MoS_2_/HfO_2_ interface (state 2) and get trapped there. Then the states 1, 1′, and 2 appear already during the forward sweep that causes an abrupt kink of *I*_D_ instead of TNDR. This also results in a sizable increase of *I*_ON_ due to self-doping with positive charges. However, since at state 2 all mobile charges are already trapped at the channel side of the oxide, they cannot move anymore, even if *V*_G_ increases, and thus the CCW hysteresis starts to localize below point 2. Finally, if the sweep is extremely slow, the state 3, followed by a kink downwards, appears at a negative *V*_G_ during the reverse sweep. This indicates the drift of mobile ions back to the gate side of HfO_2_. Furthermore, as illustrated by our additional simulation results (see Fig. S4 in the SI), the TNDR effect is not present if *N*_mob_ is decreased and becomes more pronounced if it is increased.

In Fig. 4c, we show the lower UHFs extracted from the *I*_D_–*V*_G_ curves simulated using different *E*_A_. Just like in Fig. 3b, e, a distinct maximum of the CCW hysteresis is present at the sweep frequency roughly corresponding to 1/*τ*_drift_ that is required for ions to cross *d*_ox_, i.e., if state 2 is reached at ${V}_{{{\rm{Gmax}}}}$. At the same time, a larger migration barrier obviously shifts the maximum to the slower frequencies and vice versa. In Fig. S5 in the SI, we also show that if the pre-factor of the diffusion coefficient *D*_0_ is made smaller, the maximum also moves to slower frequencies since the ions become slower and need more time to cross the oxide thickness. Based on these observations, it is clear that the same hysteresis dynamics could be obtained with different combinations of the three key parameters (see Fig. S6 in the SI). This would obviously make it complicated to use precise fits of the experimental data for the extraction of ion parameters. However, we can consider that the *E*_A_ values which we are using provide a reasonable estimate since any change for more than 0.2 eV would either result is a very different dynamics as compared to our experimental observations, or in non-physical values of *N*_mob_ and *D*_0_.

#### Boosting the TNDR magnitude with narrower sweep ranges

**Fig. 5:**
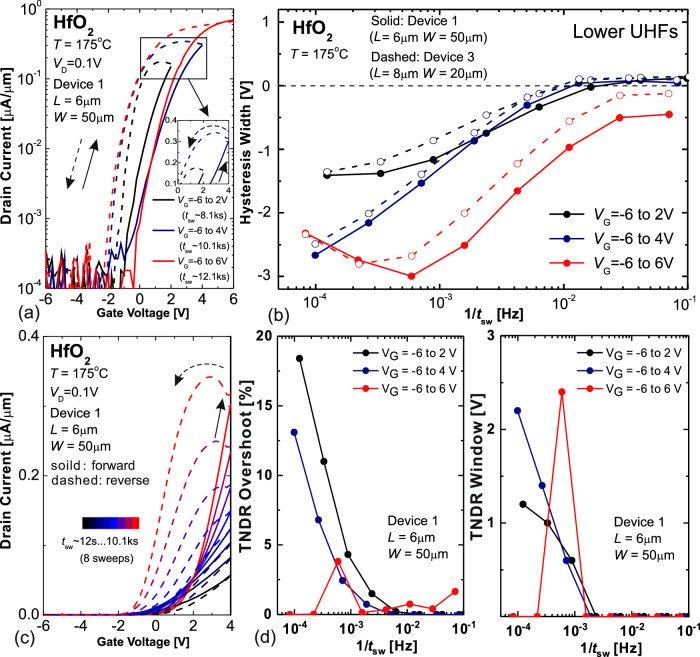
Enhancing transient negative differential resistance (TNDR) with narrower gate bias sweep ranges. **a** Double sweep *I*_D_–*V*_G_ characteristics of the MoS_2_/HfO_2_ field-effect transistor (FET) measured using the slowest achieved *t*_sw_ for different *V*_G_ sweep ranges, and *T* = 175 °C. While for −6 to 6 V the counterclockwise (CCW) hysteresis already starts to localize, for narrower sweep ranges the TNDR effect is still present. **b** The lower universal hysteresis functions (UHFs) obtained for two devices indeed show that for −6 to 6 V the maximum of CCW hysteresis is reached faster, which is because with more positive ${V}_{{{\rm{Gmax}}}}$ mobile charges need less time to cross *d*_ox_ and thus can localize at the channel side of HfO_2_ earlier. **c** Full set of double sweep *I*_D_–*V*_G_ curves measured using −6 to 4 V sweep range in a linear scale. Sizable and progressive TNDR effect towards slower sweeps is visible. **d** Frequency dependence of the TNDR overshoot *R* and TNDR window Δ*V*_TNDR_ for different gate voltage sweep ranges, revealing that narrower sweep ranges exhibit stronger TNDR effects that emerge at slower sweep rates.

Being equipped with a fundamental understanding of TNDR behavior in our MoS_2_/HfO_2_ FETs from the compact model, we next target to confine these memory dynamics by adjusting the *V*_G_ sweep range. In Fig. 5a, we show the double sweep *I*_D_–*V*_G_ characteristics of the MoS_2_/HfO_2_ FET measured at *T* = 175 °C using the slowest achieved *t*_sw_ and different *V*_G_ sweep ranges. Remarkably, while for the sweep range of −6 to 6 V the CCW hysteresis starts to localize, for the sweeps with smaller ${V}_{{{\rm{Gmax}}}}$ we still observe the TNDR effect within this sweep time range. This is a very intuitive observation, since if ${V}_{{{\rm{Gmax}}}}$ is smaller, mobile charges need more time to cross the *d*_ox_ and achieve the state 2 shown in Fig. 4a, b. The lower UHFs obtained for this and another device provided in Fig. 5b indeed confirm that for narrower sweep ranges, the maximum of the CCW hysteresis would be reached at considerably slower sweep frequencies. The full hysteresis mapping results for device 1 can be found in Fig. S7 in the SI. They particularly illustrate that due to the slower drift of mobile charges for the sweep ranges of −6 to 2 V and −6 to 4 V, a purely CW hysteresis caused by charge trapping is still observed for faster sweeps.

In Fig. 5c, we show the full set of double sweep *I*_D_–*V*_G_ characteristics measured using a −6 to 4 V sweep range in a linear scale. Remarkably, the magnitude of TNDR is considerably stronger as compared to the −6 to 6 V sweep range (e.g., Fig. 3). In Fig. 5d, we present the frequency dependence of two explicitly defined TNDR metrics: the normalized TNDR overshoot *R* (left panel) and the absolute TNDR window Δ*V*_TNDR_ (right panel). The overshoot $R=100\left(\frac{{I}_{{{\rm{peak}}}}}{{I}_{{{\rm{start}}}}}-1\right)$ quantifies the relative enhancement of the peak drain current during the reverse sweep, normalized to the initial current *I*_start_ at the last point of the forward sweep. The TNDR window Δ*V*_TNDR_ ≥ 0 denotes the absolute voltage range over which the reverse-sweep current remains above *I*_start_. We can see that both metrics exhibit a clear frequency-dependent trend across different *V*_G_ sweep ranges. For a wider sweep range of −6 to 6 V, the TNDR overshoot and window peak at moderate sweep frequencies. However, narrower sweep ranges yield a stronger effect that persists across a broader range of 1/*t*_sw_ but peaks at slower sweep frequencies. These observations align with the compact model interpretation shown in Fig. 4a, b. Specifically, when using a smaller ${V}_{{{\rm{Gmax}}}}$, we observe the high-mobility TNDR effect driven by free motion of mobile charges in the bulk of HfO_2_. In this regime, ions are largely unaffected by interface effects. Thus, the memory-like TNDR behavior (manifested as a strong overshoot and sustained window) is observed across a broader range of 1/*t*_sw_ but requires longer sweeps to get activated. The magnitude of the TNDR overshoot is primarily determined by the proximity of charges to the channel, and increases monotonically with *t*_sw_ until states 1′ and 2 are reached. However, when ${V}_{{{\rm{Gmax}}}}$ is more positive, ions cross HfO_2_ too rapidly, and we observe the low-mobility TNDR effect associated with much slower near-interface ion motion (state 1${\prime}$′). This type of TNDR behavior only appears in a narrow *t*_sw_ range, as a higher ${V}_{{{\rm{Gmax}}}}$ allows the system to reach state 2 too quickly, rendering charges immobile and suppressing both the TNDR overshoot and the TNDR window. The −6 to 4 V sweep range represents an optimal balance as it provides sufficient gate bias to drive significant charge displacement (yielding a large TNDR window) while avoiding trapping of mobile charges near the MoS_2_ interface as needed to sustain the TNDR overshoot across a wide range of operating speeds. Remarkably, the same physical picture applies to oxide thickness scaling since the drift time ${\tau }_{{{\rm{drift}}}}\propto {d}_{{{\rm{ox}}}}^{2}$. This suggests that just like a higher ${V}_{{{\rm{Gmax}}}}$ (${\tau }_{{{\rm{drift}}}}\propto 1/{V}_{{{\rm{Gmax}}}}$), thinner HfO_2_ would also shift the maxima of CCW hysteresis and TNDR metrics to faster sweep frequencies. In Fig. S8 in the SI, we demonstrate that the key trends related to the impact of *V*_G_ sweep range shown in Fig. 5, as well as the temperature dependence, can be qualitatively captured by our compact model. Furthermore, in Fig. S9, we demonstrate that TNDR behavior and its key metrics show reasonable cycle-to-cycle reproducibility for over 30 consecutively measured *I*_D_–*V*_G_ characteristics.

We note that classification of the observed TNDR mechanisms based on our experimental results and compact model may be useful for future design and precise control of memory dynamics in 2D FETs. For instance, the impact of oxide thickness, interface quality, and intentional doping with mobile impurities other than preexisting oxygen vacancies could be discovered.

### MoS_2_/Al_2_O_3_ FETs at high temperatures: superior stability

**Fig. 6:**
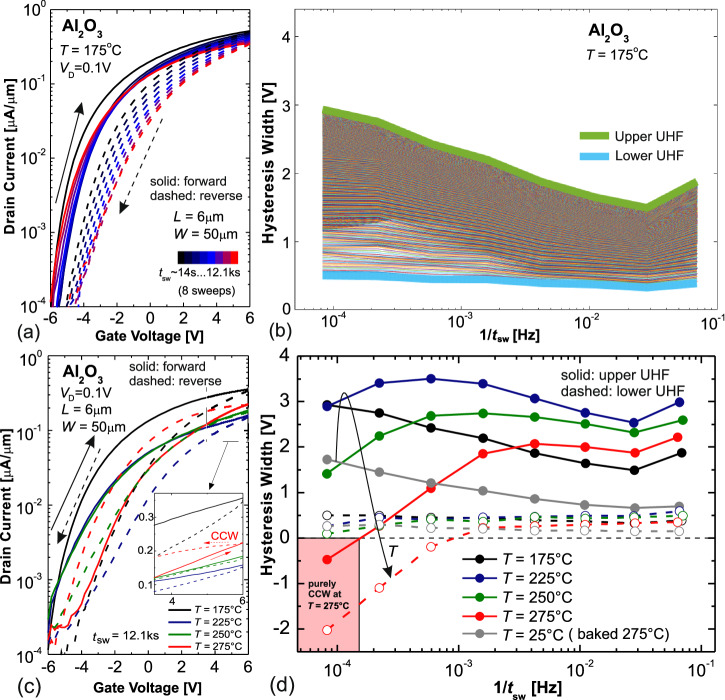
High-temperature stability of MoS_2_/Al_2_O_3_ transistors. **a** Double sweep *I*_D_–*V*_G_ characteristics of our MoS_2_/Al_2_O_3_ field-effect transistor (FET) measured at *T* = 175 °C using 8 subsequent sweeps with *t*_sw_ up to 12.1 ks. **b** The corresponding mapping results showing that the hysteresis remains purely clockwise (CW) just like it was at room temperature. **c** Double sweep *I*_D_–*V*_G_ characteristics measured for the same device up to *T* = 275 °C  using the slowest achieved *t*_sw_. Transition to the counterclockwise (CCW) hysteresis at higher temperatures accompanying with slight self-doping, is clearly visible. **d** The corresponding upper and lower universal hysteresis functions (UHFs) showing change from thermal activation of charge trapping up to *T* = 225 °C to activation of the CCW mechanism related to the drift of oxygen vacancies at higher temperatures. The post-annealing *T* = 25 °C  curves that show smaller and purely CW hysteresis nicely match the trends.

In Fig. 6a, we show the double sweep *I*_D_–*V*_G_ characteristics of our MoS_2_/Al_2_O_3_ FET measured at *T* = 175 °C using 8 subsequent sweeps with *t*_sw_ up to 12.1 ks. The corresponding hysteresis mapping results provided in Fig. 6b confirm that, just like it was at room temperature, purely CW hysteresis is present. However, the results measured for the same device up to *T* = 275 °C (Fig. 6c, d) still allowed us to catch the interplay between the CW and CCW mechanisms (for better understanding, see the full mapping results for all temperatures in Fig. S10 in the SI). Namely, up to *T* = 225 °C, we are dealing with thermally activated charge trapping by oxide traps in Al_2_O_3_. This results in a well-known decrease of *I*_D_ at slow sweeps (Fig. 6c and inset) and a classical bell-shape maximum of the CW hysteresis that shifts to faster frequencies vs. temperature^19^. Being rarely observed for amorphous oxides with broad defect bands, here this CW maximum is nicely captured within our *t*_sw_ range at *T* = 225 °C (blue curve in Fig. 6d). However, at *T* = 250 °C thermal activation of mobile charges starts that partially compensates the left part of the CW maximum, and at *T* = 275 °C we finally see a purely CCW hysteresis at slow sweeps that comes together with the current increase due to self-doping, as was discussed above for the devices with HfO_2_. Remarkably, the *T* = 275 °C results for this MoS_2_/Al_2_O_3_ FET are very similar to those measured for our MoS_2_/HfO_2_ device at *T* = 125 °C  (see Fig. S3a, b in the SI). This suggests that the origin of the CCW hysteresis in both devices is similar, being related to the drift of oxygen vacancies. However, in Al_2_O_3_, the same CCW effects start to appear for the temperatures that are higher by at least 150 °C. Based on our compact model, this suggests that Al_2_O_3_ should have *E*_A_ for migration of oxygen vacancies of about 1.6 eV as compared to 1.15 eV that we assumed for HfO_2_. This may be because ionic Al-O bonds are stronger as compared to Hf-O bonds. Therefore, these results clearly show that Al_2_O_3_ enables far better stability with respect to the CCW hysteresis caused by the drift of oxygen vacancies as compared to HfO_2_, though at the same time being less relevant to achieve TNDR memory performance. We also note that the observed high-temperature hysteresis dynamics are not affected by gate leakage or dielectric breakdown, as confirmed by the gate leakage characteristics (*I*_G_–*V*_G_) measured for both MoS_2_/HfO_2_ and MoS_2_/Al_2_O_3_ FETs at elevated temperatures during long hysteresis sweeps (see Fig. S11 in the SI).

### Bias stress stability of Al_2_O_3_ and HfO_2_ at high temperatures

**Fig. 7:**
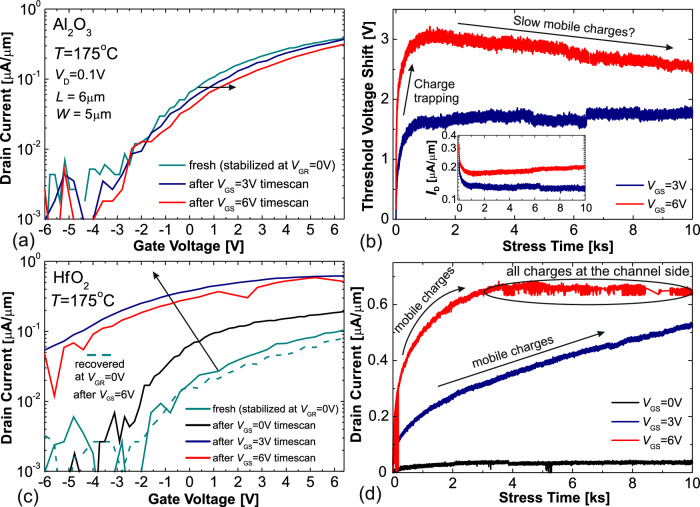
High-temperature bias stress stability. **a** Reference *I*_D_–*V*_G_ characteristics of our MoS_2_/Al_2_O_3_ field-effect transistor (FET) measured before and after *t*_s_ = 10 ks gate bias stresses. **b** The Δ*V*_th_ vs. *t*_s_ dependences recalculated from the *I*_D_ traces shown in the inset. An increase of positive Δ*V*_th_ caused by charge trapping is observed, with possible minor signs of reversal for *V*_GS_ = 6 V starting from 1 ks that should be due to activation of slow oxygen vacancies in Al_2_O_3_. **c** Reference *I*_D_–*V*_G_ characteristics of the MoS_2_/HfO_2_ FETs measured before and after *t*_s_ = 10 ks gate bias stresses reveal a sizable negative shift of Δ*V*_th_ that is due to the drift of positive charges to the channel side of HfO_2_. **d** The *I*_D_ vs. *t*_s_ traces clearly show a strong current increase, with possible saturation for *V*_GS_ = 6 V when all mobile vacancies reach the channel side of HfO_2_. Extraction of Δ*V*_th_ is barely possible in this case, though its negative sign is obvious.

To solidify the above findings obtained using hysteresis analysis, we finally examine our MoS_2_/Al_2_O_3_ and MoS_2_/HfO_2_ FETs under constant bias stress at *T* = 175 °C . These tests focused on tracking the time evolution of *I*_D_ up to *t*_s_ = 10 ks at subsequently increased positive stress voltages *V*_GS_ following 10 ks stabilization at *V*_GR_ = 0 V, while doing fast control *I*_D_–*V*_G_ sweeps before and after each stress. The results for our MoS_2_/Al_2_O_3_ devices are shown in Fig. 7a, b. Using the fresh reference *I*_D_–*V*_G_ curve obtained after initial device stabilization (Fig. 7a), we can recalculate the measured *I*_D_(*t*) traces (Fig. 7b, inset) into the Δ*V*_th_(*t*) dependences (Fig. 7b). These results confirm that generally we are dealing with positive Δ*V*_th_ that originates from charge trapping by oxide traps and causes a decrease of *I*_D_. However, the curves obtained using *V*_GS_ = 6 V indicate a clear reversal in the trend with some increase of *I*_D_ and thus partial compensation of Δ*V*_th_ starting at about 1 ks. This suggests activation of slow mobile oxygen vacancies in Al_2_O_3_. As was discussed above, they should have larger *E*_A_ as compared to the same defects in HfO_2_ and thus cannot be captured in hysteresis measurements as the CCW trends would have appeared at inaccessibly slow *t*_sw_. Indeed, as illustrated in Fig. 6, they introduce the CCW contribution of hysteresis at higher temperatures. We also note that the Δ*V*_th_ values measured using a constant current mode (Fig. 7b) are considerably larger as compared to the ones that we could get by comparing the reference curves (Fig. 7). This suggests that the degradation partially recovers already during a few seconds of the control *I*_D_–*V*_G_ sweeps that is typical for oxide traps at high temperatures^36^.

The related results for our MoS_2_/HfO_2_ FETs, provided in Fig. 7c, d, show a totally different behavior which matches the CCW hysteresis dynamics discussed above. Already from the reference curves (Fig. 7c), we see large negative Δ*V*_th_ after positive bias stresses that come together with overall increase of *I*_D_. The *I*_D_(*t*) traces (Fig. 7d) also show a strong current increase that becomes faster and reaches saturation with *V*_GS_ = 6 V. The latter indicates that all charges have reached their equilibrium positions at the channel side of HfO_2_ (i.e., state 2 in Fig. 4b). However, no recalculation into the Δ*V*_th_(*t*) dependences is possible since the vertical drifts and transformation of the shapes of *I*_D_–*V*_G_ curves make any precise definition of Δ*V*_th_ impossible. Still in Fig. S12 in the SI, we demonstrate that the obtained *I*_D_(*t*) traces can be qualitatively captured using our compact model for mobile charges with the parameters similar to those used for hysteresis. Furthermore, observations of Fig. 7c, such as degradation of SS in the post-stress curves and complete recovery after 10 ks at *V*_GR_ = 0 V, nicely complement our findings. The former effect can be explained by remote scattering at the positive charges coming closer to the MoS_2_/HfO_2_ interface, and the latter one should be due to the return of mobile charges back to the gate side of HfO_2_.

## Conclusions

In summary, we have performed a detailed comparison of hysteresis dynamics and bias stress stability in MoS_2_/HfO_2_ and MoS_2_/Al_2_O_3_ FETs fabricated using the same process up to 275 °C. Our initial findings indicate that room temperature stability limitations for both device types originate from oxide traps that cause CW hysteresis, with Al_2_O_3_ showing a slight advantage due to a more favorable fundamental defect band alignment. However, our major results demonstrate that at higher temperatures, the devices with HfO_2_ exhibit additional severe instabilities, which appear as the CCW hysteresis and negative *V*_th_ drift under a positive gate bias. This behavior and accompanying *I*_D_ increase with possible TNDR features can be nicely described by our compact model for mobile charges. The positive oxygen vacancies (${{{\rm{V}}}}_{{{\rm{O}}}}^{+}$ or ${{{\rm{V}}}}_{{{\rm{O}}}}^{2+}$) in HfO_2_ having migration barriers of about 1 eV are suggested as most likely candidates. Since in Al_2_O_3_ these vacancies are expected to be slower due stronger ionic bonds, for our MoS_2_/Al_2_O_3_ FETs, the same effect starts to be pronounced in hysteresis measurements only at 275 °C. Our results reveal an essential insulator selection paradigm for the development and integration of 2D FET technology: while Al_2_O_3_ is superior to suppress negative *V*_th_ drifts for high-temperature logic applications, their HfO_2_ counterparts can serve as functional active layers that leverage these instabilities to enable intrinsic memory functionality. The latter would require additional device engineering efforts, such as adjusting the device structures or tuning the concentrations of mobile charges in gate insulators.

## Methods

Device fabrication: MoS_2_/HfO_2_ and MoS_2_/Al_2_O_3_ FETs were fabricated using an identical process employing commercial CVD-grown monolayer MoS_2_ films taken from the same batch. Their fabrication process was arranged as follows. First, the Si/SiO_2_ substrates were ultrasonically cleaned in acetone and alcohol for 10 min each to remove surface contaminants. Local back-gate patterns were then defined via photolithography on the pretreated Si/SiO_2_ substrates, followed by deposition of a 10 nm Ni/30 nm Au metal stack as the back-gate electrode using e-beam evaporation. Next, a ~20 nm-thick insulator (HfO_2_ or Al_2_O_3_) was grown via ALD under optimized process parameters (substrate temperature of 250 °C), guaranteeing the homogeneity and interfacial flatness of the insulator. Subsequently, the MoS_2_ films were precisely transferred onto the insulator surface via a PMMA-assisted wet transfer method. Residual polymers were removed by acetone and alcohol soaking, followed by N_2_ blow-drying. The MoS_2_ channels were patterned via photolithography and reactive ion etching. Finally, the source/drain contact regions were precisely defined by photolithography. 10 nm Ni/30 nm Au was deposited via e-beam evaporation followed by a standard lift-off process, thus completing the back-gate device fabrication.

Electrical characterization: electrical characterization of MoS_2_/HfO_2_ and MoS_2_/Al_2_O_3_ FETs was conducted in a vacuum probe station (HCP-O-2, TIANHENG KEYI (SUZHOU) OPTOELEC TECH CO., LTD) with a base pressure of ~5 × 10^−6^ torr. All measurements were performed in complete darkness over a temperature range from 25 to 275 °C. For electrical measurements, we used a Keithley 4200A-SCS semiconductor parameter analyzer controlled by a lab-built Python graphical interface to enable uninterrupted long-term testing. Hysteresis dynamics were characterized via double-sweep *I*_D_–*V*_G_ measurements with a voltage step of 0.2 V. The sweep time *t*_sw_ was varied from a few seconds to about 12 ks, and the gate voltage sweep ranges included −6 to 2 V, −6 to 4 V, and −6 to 6 V. The bias stress analysis was performed by measuring *I*_D_ vs. *t*_s_ traces up to 10 ks using *V*_GS_ gradually increased from 0 to 6 V, while applying 10 ks stabilizing rounds with *V*_GR_ = 0 V and doing control *I*_D_–*V*_G_ sweeps before each stressing round. Using the initially measured reference *I*_D_-*V*_G_ curve, we converted these results into Δ*V*_th_(*t*_s_) dependences for the MoS_2_/Al_2_O_3_ FETs and concluded that it is not possible for the MoS_2_/HfO_2_ devices due to non-parallel drifts caused by *I*_D_ increase.

Extraction of the hysteresis width: the hysteresis width Δ*V*_H_ is defined as the distance between forward and reverse sweep *I*_D_–*V*_G_ curves in the *V*_G_ axis, being positive for CW and negative for CCW hysteresis. To properly extract Δ*V*_H_ from the measured *I*_D_–*V*_G_ vs. *t*_sw_ datasets, we applied our universal hysteresis mapping method^27^. This approach suggests scanning from the current value slightly above *I*_OFF_ to near-*I*_ON_ values as opposed to a single constant current extraction used in our previous works^19,28^. The exact mapping range was slightly adjusted depending on the presence of obvious artifacts in a particular dataset, for instance, noise at low currents. Using a very fine step on ${I}_{{{\rm{Dconst}}}}$ (i.e., 3000 points), we obtained a series of Δ*V*_H_(1/*t*_sw_) curves. Then, in our raw analysis, we expressed the hysteresis dynamics as a distribution of these curves, concluded in between the upper and lower UHFs constructed as a piecewise maximum and minimum from the obtained set of Δ*V*_H_(1/*t*_sw_) dependences, respectively. A purely CW hysteresis is realized if both UHFs are positive, making it feasible to operate only with the upper UHFs. If both UHFs are negative, the hysteresis is purely CCW and can be described with the lower UHF. Finally, if the upper UHF is positive and the lower UHF is negative, the CW/CCW switching is present, and both UHFs are needed to describe the hysteresis correctly. The same also applies to the time separation of the CW/CCW hysteresis when UHFs change their signs vs. 1/*t*_sw_.

Compact model: our compact model describes thermally activated hopping of mobile charges in the oxide with the diffusion coefficient $D={D}_{0}\exp \left(-\frac{q{E}_{{{\rm{A}}}}}{{k}_{{{\rm{B}}}}T}\right)$ defined by the activation barrier *E*_A_ and constant pre-factor *D*_0_. Using this diffusion coefficient, we obtain the ion mobility from the Einstein relation and subsequently use it to get their drift velocity under applied gate bias. Next, we set the equilibrium positions of positive mobile charges at the gate and channel sides of the oxide for negative and positive *V*_G_, respectively, and apply an iterative scheme to calculate the time-dependent positions of ions *x*(*t*) during the sweeps. Finally, knowing *x*(*t*), we calculate the resulting Δ*V*_th_ caused by the drift of mobile charges, and use it for the calculation of *I*_D_–*V*_G_ curves for different *t*_sw_. The model setup is implemented in MATLAB. Full description with all equations used can be found in the SI.

## Supplementary information

Supporting Info

Transparent Peer Review File

## Data Availability

The data that support the findings of this study are available from the corresponding authors upon reasonable request.
