# Supplementary material for: Mobile charges in MoS2/high-k oxide transistors: from abnormal instabilities to transient negative differential resistance
Source: Commun Eng. 2026 Jul 2;5:120. doi: 10.1038/s44172-026-00716-2 (PMC13333013; doi:10.1038/s44172-026-00716-2)
Supplement: Supplementary file 1 — Supporting Info [file 44172_2026_716_MOESM1_ESM.pdf]

Mobile charges in MoS_2/high-k oxide transistors: from abnormal instabilities to transient negative differential resistance (Supplementary Information)

Shaokai Zhou,[†] Haihui Cai,[†] Yehao Wu,[†] Yufeng Min,[‡] Renchen Yuan,[‡] Yezhu Lv,[†] Jianming Huang,[†] Yuanyuan Shi,[,‡] and Yury Yuryevich Illarionov[*,†]*

†*Laboratory of 2D Optoelectronics and Nanoelectronics (L2DON), State Key Laboratory of Quantum Functional Materials, Department of Materials Science and Engineering, Southern University of Science and Technology, 1088 Xueyuan Blvd, 518055 Shenzhen, China*

‡*School of Microelectronics, University of Science and Technology of China, 230026 Hefei, China*

E-mail: yuanyuanshi@ustc.edu.cn; illarionov@sustech.edu.cn

Full mapping results for hysteresis measured at 25°C

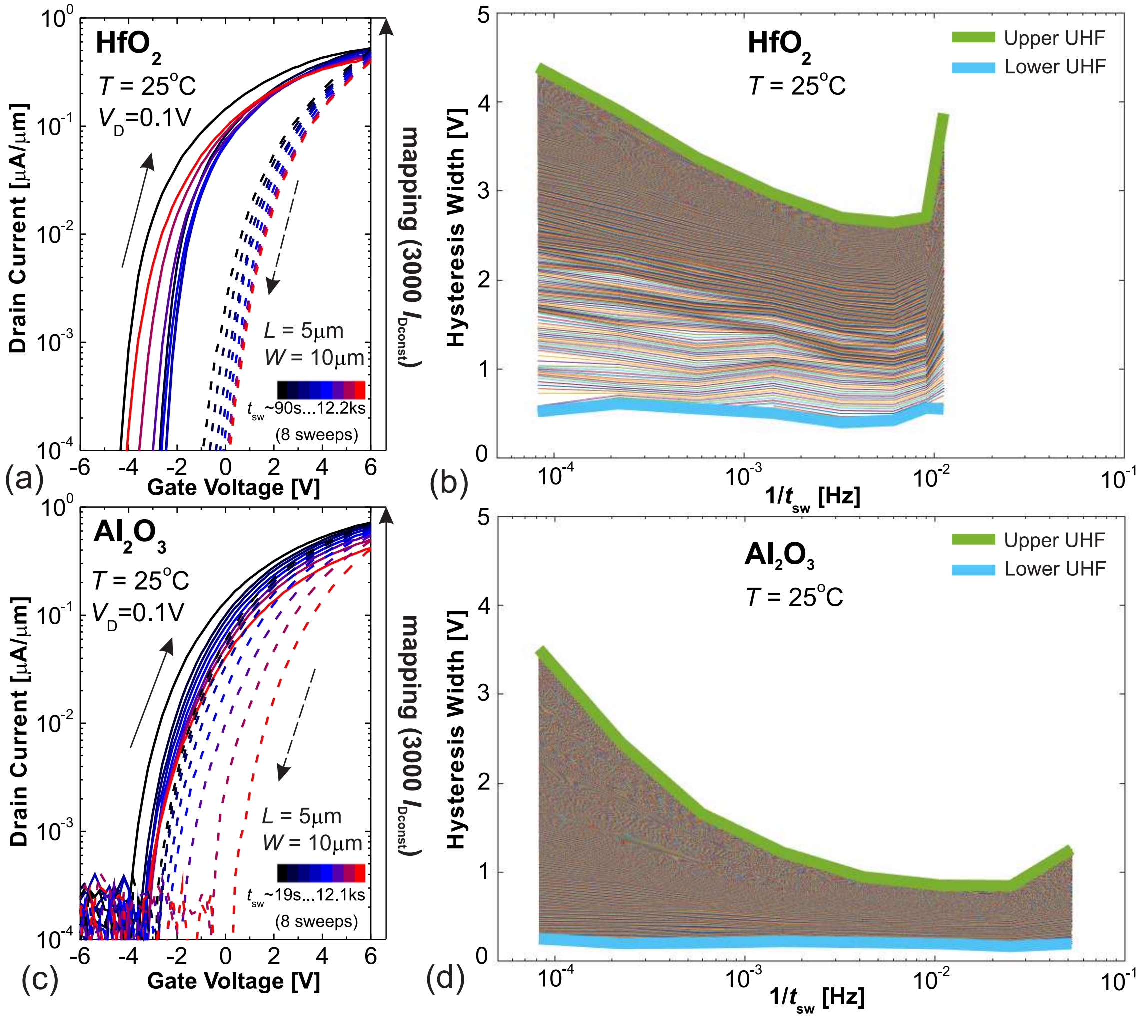

Figure S1: **Full mapping results for room-temperature hysteresis.** (a) Double sweep I_D-V_G characteristics measured using 8 subsequently increased t_{sw} for our MoS_2/HfO_2 FET. (b) The corresponding mapping results showing purely CW hysteresis. (c,d) The related results for a representative MoS_2/Al_2O_3 FET with the same dimensions. A smaller but still purely CW hysteresis is revealed.

In Fig.S1 we show the full hysteresis mapping results for the representative MoS_2/HfO_2 and MoS_2/Al_2O_3 FETs with the same channel dimensions. By using the measurement datasets consisting of 8 subsequent I_D-V_G sweeps with t_{sw} of up to about 12 ks (Fig.S1a,c), we scan 3000 constant current points and extract series of $\Delta V_H(1/t_{sw})$ curves. This allows us to obtain the upper and lower UHFs as shown in Fig.S1b,d. Since in these particular

cases the hysteresis is purely CW, only the upper UHFs present practical interest for further analysis. At the same time, consistent representation of the final $\Delta V_\mathrm{H}(1/t_\mathrm{sw})$ dependence as the upper UHF is still of key importance since it is far more accurate as compared to the use of just a single randomly selected I_Dconst value. As a result, reasonable comparison of hysteresis dynamics measured for different devices becomes possible. For instance, it is obvious that in our case smaller hysteresis in MoS_2/Al_2O_3 FETs as compared to their MoS_2/HfO_2 counterparts is related to the device properties rather than extraction methodology.

Additional results for the temperature dependence of hysteresis in MoS_2/HfO_2 FETs

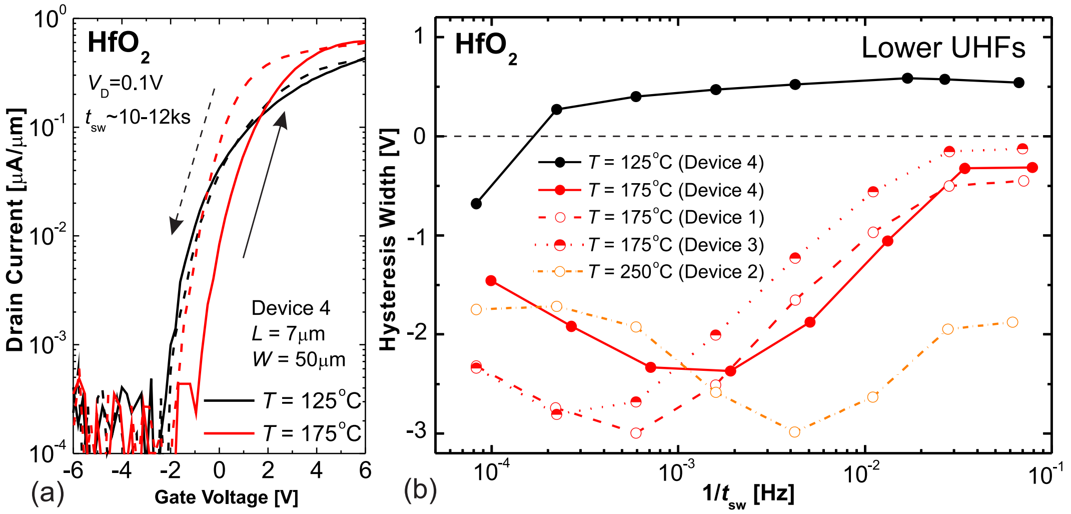

Figure S2: **High-temperature hysteresis in different MoS_2/HfO_2 devices.** (a) Double sweep I_D-V_G characteristics of the same MoS_2/HfO_2 FET measured at $T = 125$°C and 175°C using slow sweeps. At 125°C only minor signs of the CCW hysteresis appear since mobile charges are too slow. (b) The corresponding lower UHFs (solid lines) confirm strong thermal activation of mobile charges at 175°C and 250°C. This compensates the CW hysteresis coming from charge trapping that is still dominant at 125°C. The 175°C curves for two additional devices (dashed and dotted lines) illustrate that the trends in CCW hysteresis are well reproducible.

In Fig.S2a we show the double sweep I_D-V_G characteristics of the same MoS_2/HfO_2 FET measured at $T = 125$°C and 175°C using slow sweeps. As confirmed by the corresponding

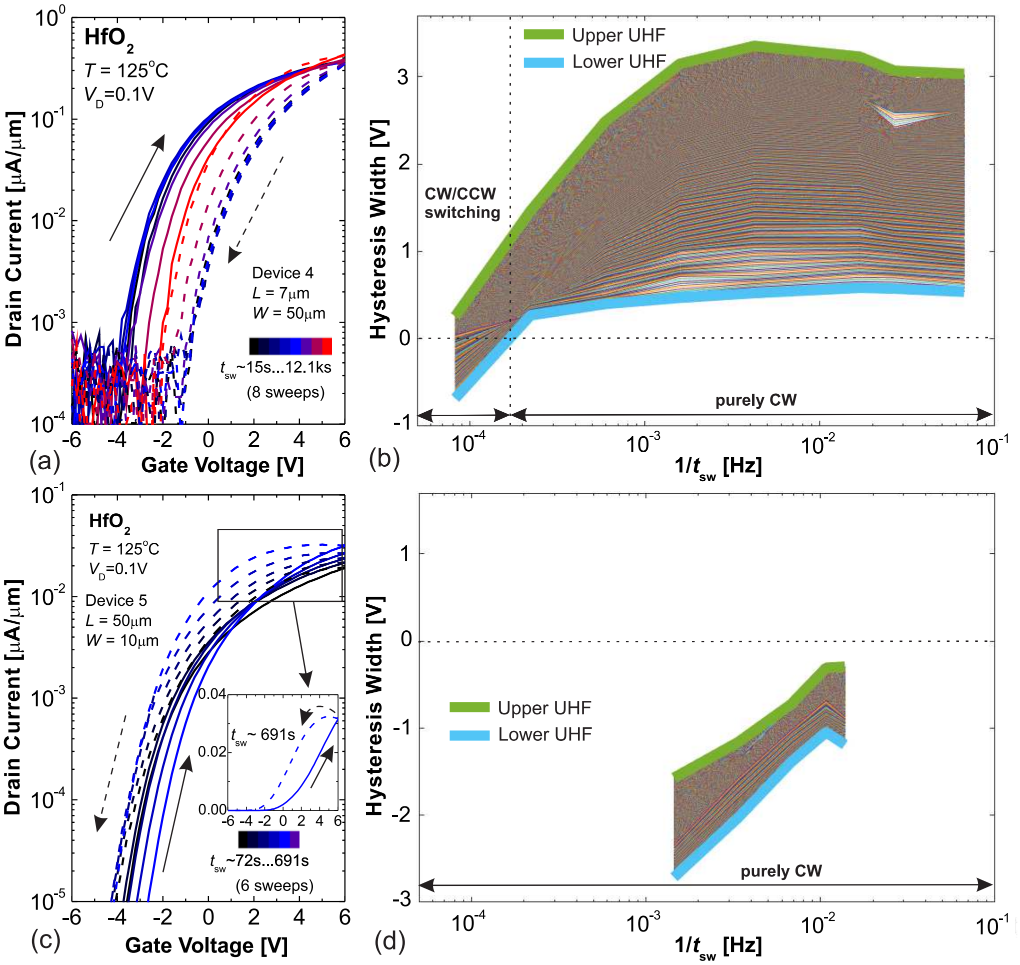

Figure S3: **Additional hysteresis results measured at $T = 125°C$.** (a) Double sweep I_D-V_G characteristics of our MoS_2/HfO_2 FET measured at $T = 125°C$ using 8 subsequent sweeps with t_{sw} up to 12.1 ks. (b) Full hysteresis mapping results showing the dominant CW hysteresis with some CW/CCW switching only at slow sweeps. (c,d) More fragmentary results for another device measured at $T = 125°C$ 3 months earlier which show purely CCW hysteresis with TNDR at relatively fast sweeps.

lower UHFs in Fig.S2b, at $T = 125°C$ the CW hysteresis is still dominant, with only some CW/CCW switching coming into play at about 10 ks sweep time. This indicates that mobile oxygen vacancies are still slow. However, 175°C results for 3 devices showing a distinct maximum of the CCW hysteresis clearly illustrate that this temperature is sufficient to activate mobile charges, and at 250°C the drift of mobile charges is accelerated further.

In Fig.S3a,b we show the full set of I_D-V_G characteristics and the corresponding mapping

results measured for the Device 4 from Fig.S2 at $T = 125$°C. As the mobile charges are much slower than at 175°C, the CW hysteresis caused by charge trapping is dominant and some CW/CCW switching appears only at very slow sweeps. However, another device measured 3 months earlier also at $T = 125$°C (Fig.S3c,d) shows purely CCW hysteresis with TNDR effects at relatively fast sweeps. According to the modeling results provided in this work, this suggests that the concentration of mobile charges N_{mob} in that device was much higher. However, subsequent storage of the device could result in partial substitution of oxygen vacancies so that in later measurements the same effects with CCW hysteresis become dominant only at higher temperatures. At the same time, some variability in N_{mob} due to different local quality of HfO_2 may be another reason for the observed difference.

Compact model for the CCW hysteresis caused by positive mobile charges

The core physical approach of our compact model is to link the ion mobility in the oxide and the threshold voltage of a MoS_2 FET through a position-dependent electrostatics. This is done by using the following key steps which are implemented into our Matlab script.

Step 1: Incorporate thermally activated hopping of mobile charges

The diffusion coefficient for thermally activated hopping of mobile charges is given as

$$D = D_0 \exp\left(-\frac{qE_\mathrm{A}}{k_\mathrm{B}T}\right) \tag{1}$$

where D_0 is a pre-factor coefficient and E_A is the energy activation barrier for the migration of ions. Both these parameters play the key role in defining the dynamics of mobile charges in the oxide.

Step 2: Obtain the mobility of ions in the oxide from the Einstein relation

The Einstein relation links the diffusion coefficient and mobility of a charged particle. Then we can get the following equation for mobility of ions in the oxide

$$\mu = \frac{qD}{k_\mathrm{B}T} = \frac{q}{k_\mathrm{B}T}\left[D_0 \exp\left(-\frac{qE_\mathrm{A}}{k_\mathrm{B}T}\right)\right] \tag{2}$$

This makes it possible to convert the random diffusion process into directed drift under electric field.

Step 3: Obtain drift velocity of mobile charges under gate bias

Knowing the mobility, we can obtain the drift velocity of ions under gate bias which can be represented as

$$v_\mathrm{drift} = \mu|E_\mathrm{ox}| = \mu\left|\frac{V_\mathrm{G}}{d_\mathrm{ox}}\right| \tag{3}$$

where the oxide electric field $E_\mathrm{ox} = V_\mathrm{G}/d_\mathrm{ox}$ provides the driving force for ion motion.

Step 4: Determine the time evolution of the position of ions

Ions move to their equilibrium positions (x_{eq}) which is set by the electric field direction in the oxide. Then we consider that under positive V_{G} the equilibrium will be near the channel interface ($x_{\text{eq}} = 0.1\, d_{\text{ox}}$), and under negative V_{G} it will be near the gate electrode ($x_{eq} = 0.9 d_{ox}$). In that case the actual rate of position change towards equilibrium can be determined as

$$\frac{dx}{dt} = \frac{x_{\text{eq}} - x}{\tau_{\text{drift}}} \tag{4}$$

with the characteristic drift time $\tau_{\text{drift}} = d_{\text{ox}}/v_{\text{drift}}$ representing the time required for ions to cross the oxide. By discretizing the analytical solution of this differential equation, we can obtain the position update of ions at every next time step as

$$dx = (x_{\text{eq}} - x_{\text{current}}) \times \Big[1 - \exp\Big(-B\frac{dt}{\tau_{\text{drift}}}\Big)\Big] \tag{5}$$

with the current position x_{current} being considered as the initial position for each step in the loop which states that $x_{\text{new}} = x_{\text{current}} + dx$. Note that considering that the sweeps start at negative V_{G}, for positive charges we use the averaged starting position $0.8 d_{\text{ox}}$ at the first step (i.e. close to the gate). The constant parameter B takes into account slowing of ions near the interfaces and their faster motion in the oxide bulk. In our qualitative model setup we use the value of 0.01 if the normalized position of ion $x/d_{\text{ox}} < 0.15$ (i.e. too close to the channel/oxide interface), 0.03 for $0.15 < x/d_{\text{ox}} < 0.3$, 5 for $0.15 < x/d_{\text{ox}} < 0.85$ and 0.02 for $x/d_{\text{ox}} > 0.85$ (i.e. too close to the gate/oxide interface). The physical meaning of $1/\tau_{\text{drift}}$ is the relaxation rate which thus can be adjusted via multiplying by the position factor B. We note that in principle the diffusion contribution coming from random motion of ions could be added to dx. However, here we assume that the drift caused by applied gate bias has the dominant impact.

Finally, physical constraints $x_{\text{new}} = \max(0.1 d_{\text{ox}}, \min(0.9 d_{\text{ox}}, x_{\text{new}}))$ are set to prevent non-

physical positions that are too close to interfaces which ions cannot occupy due to their finite sizes and interface barriers.

Step 5: Calculation of the threshold voltage shift induced by motion of mobile charges

Knowing the positions of mobile charges at every time step, we next calculate the time-dependent threshold voltage shift which will be induced by their motion in the oxide. It can be written as

$$\Delta V_{\mathrm{th}}(t) = -\frac{Q_{\mathrm{max}}}{C_{\mathrm{ox}}}\left(1 - \frac{x(t)}{d_{\mathrm{ox}}}\right) \tag{6}$$

where $Q_{\mathrm{max}} = qN_{\mathrm{mob}}d_{\mathrm{ox}}$, N_{mob} is the concentration of mobile charges in the oxide and $C_{\mathrm{ox}} = \varepsilon_{\mathrm{ox}}\varepsilon_0/d_{\mathrm{ox}}$ is the oxide capacitance. The minus sign takes into account that we consider positive charges which should create a negative ΔV_{th} that will have a maximum when all ions accumulate near the channel/oxide interface (i.e. $x = 0$).

Step 6: Calculation of the time-dependent drain current

To calculate the drain current using the obtained ΔV_{th} induced by the ion drift, we first evaluate the interface charge based on the Fermi-Dirac distribution as

$$Q_{\mathrm{it}} = -qD_{\mathrm{it}}k_{\mathrm{B}}T \ln\left[1 + \exp\left(\frac{q(V_{\mathrm{G}} - V_{\mathrm{FB}})}{k_{\mathrm{B}}T}\right)\right] \tag{7}$$

where D_{it} is the density of interface states at the channel/oxide interface and the flat band voltage is determined as

$$V_{\mathrm{FB}} = \Phi_{\mathrm{MS}} - \frac{E_{\mathrm{G}}}{2} + \frac{qD_{\mathrm{it}}E_{\mathrm{G}}}{2C_{\mathrm{ox}}} \tag{8}$$

with Φ_{MS} being the effective work function difference between the gate metal and the MoS_2 channel and E_{G} the MoS_2 bandgap. We note that Φ_{MS} is a tuning parameter that lumps together the gate work function, fixed charges and contact effects such as Fermi level pinning. This simplification is acceptable when calculating V_{FB} in a qualitative compact model.

Then the effective gate voltage which takes into account possible screening by interface charges can be obtained as

$$V_{\mathrm{G,eff}} = V_{\mathrm{G}} - \frac{Q_{\mathrm{it}}}{C_{\mathrm{ox}}} \tag{9}$$

Finally, the ion-induced ΔV_{th} calculated above can be used for piecewise calculation of the drain current as

$$I_{\mathrm{D}}(V_{\mathrm{G}}, t) = \begin{cases} I_{\min} \cdot \exp\left(\dfrac{V_{\mathrm{G,eff}} - V_{\mathrm{th}} - \Delta V_{\mathrm{th}}}{SS}\right) & V_{\mathrm{G,eff}} \leq V_{\mathrm{th}} + \Delta V_{\mathrm{th}} \\ \mu_{\mathrm{eff}} \cdot n_{2\mathrm{D}} \cdot q \cdot \dfrac{W}{L} \cdot V_{\mathrm{D}} & V_{\mathrm{G,eff}} > V_{\mathrm{th}} + \Delta V_{\mathrm{th}} \end{cases} \tag{10}$$

where the carrier density is $n_{2\mathrm{D}} = C_{\mathrm{eff}}(V_{\mathrm{G,eff}} - V_{\mathrm{th}} - \Delta V_{\mathrm{th}})/q$ with effective capacitance $C_{\mathrm{eff}} = C_{\mathrm{ox}}(C_{\mathrm{q}} + C_{\mathrm{it}})/(C_{\mathrm{ox}} + C_{\mathrm{q}} + C_{\mathrm{it}})$ accounting for the quantum capacitance effects in the MoS_2 channel via $C_{\mathrm{q}} = q^2 m_{\mathrm{eff}}/\pi\hbar^2$ and the capacitance of interface states as $C_{\mathrm{it}} = qD_{\mathrm{it}}$ with m_{eff} being the effective carrier mass in MoS_2 and D_{it} the density of interface states, respectively. The equilibrium threshold voltage V_{th} is set to be 0.5 V above the calculated V_{FB} for simplicity.

Implementation and input parameters of the model

In our Matlab implementation we set the V_{G} arrays from V_{Gmin} to V_{Gmax} and back and assign the corresponding time point from the time array of 0 to t_{sw} to each V_{G} point. This obviously makes the results dependent on the input t_{sw} and the V_{Gmin} to V_{Gmax} sweep range so that the hysteresis dynamics observed experimentally could be reproduced qualitatively.

The input material-related parameters of the MoS_2/HfO_2 FETs include the insulator thickness $d_{\mathrm{ox}} = 20\,\mathrm{nm}$, the permittivity of HfO_2 $\varepsilon_{\mathrm{ox}} = 20$, the electronic bandgap of MoS_2 $E_{\mathrm{G}} = 2.53\,\mathrm{eV}$, the effective electron mass in MoS_2 $m_{\mathrm{eff}} = 0.45\,m_0$ and the effective work function difference $\Phi_{\mathrm{MS}} = -0.2\,\mathrm{eV}$ selected to position the subthreshold region similarly to the experimental I_{D}-V_{G} curves.

For the simplicity, we also input the minimum current in the OFF state $I_{\min} = 10^{-13}\,\mathrm{A}$,

effective carrier mobility in MoS_2 $\mu_{\mathrm{eff}} = 10\,\mathrm{cm^2V^{-1}s^{-1}}$ and the subthreshold swing $\mathrm{SS} = 280\,\mathrm{mV/dec}$ to the equation (10). The density of interface states $D_{\mathrm{it}} = 2\times10^{12}\,\mathrm{cm^{-2}eV^{-1}}$ is used to consider possible acceptor-type fixed charges at the channel/oxide interface. The drain voltage $V_{\mathrm{D}} = 0.1\,\mathrm{V}$ is set just like in our experiments, as well as the representative channel length $L = 6\,\mu\mathrm{m}$ and width $W = 50\,\mu\mathrm{m}$.

Finally, the key parameters which determine the dynamics of ion drift in the oxide are the total concentration of mobile charges N_{mob}, the activation energy of their migration E_{A} and the constant pre-factor D_0 which represents the maximum possible diffusion coefficient if the activation barrier is removed. The best qualitative agreement with our experimentally observed hysteresis dynamics is obtained using $N_{\mathrm{mob}} = 3\times10^{19}\,\mathrm{cm^{-3}}$, $E_{\mathrm{A}} = 1.15\,\mathrm{eV}$ and $D_0 = 2\times10^{-7}\,\mathrm{m^2/s}$. However, for the proof-of-concept demonstrations we also changed these values to illustrate the key trends as specified in the figures.

Impact of N_{mob} and D_0 on the CCW hysteresis dynamics from the compact model

In Fig.S4a we show the double sweep I_{D}-V_{G} characteristics of the MoS_2/HfO_2 FET simulated with our compact model considering a reduced $N_{\mathrm{mob}} = 3\times10^{18}\,\mathrm{cm^{-3}}$. For this minor concentration of mobile charges in HfO_2, the CCW hysteresis is small while the self-doping is insufficient to cause any TNDR effect or sizable increase of the ON current. However, an obvious kink between the points 1 and 2 is still visible for slow sweeps. This indicates that migration of positive charges to the channel side of HfO_2 still takes place, even though their concentration is small. In contrast, the results simulated with higher $N_{\mathrm{mob}} = 3.6\times10^{19}\,\mathrm{cm^{-3}}$ (Fig.S4b) reveal a strong TNDR effect at moderate sweeps, as well as a more pronounced increase of I_{ON} which at slow t_{sw} turns into a large and abrupt kink between the points 1 and 2. In Fig.S4c we show the lower UHFs extracted from the I_{D}-V_{G} curves simulated using different N_{mob}. It is obvious that for larger concentrations of ions the CCW hysteresis

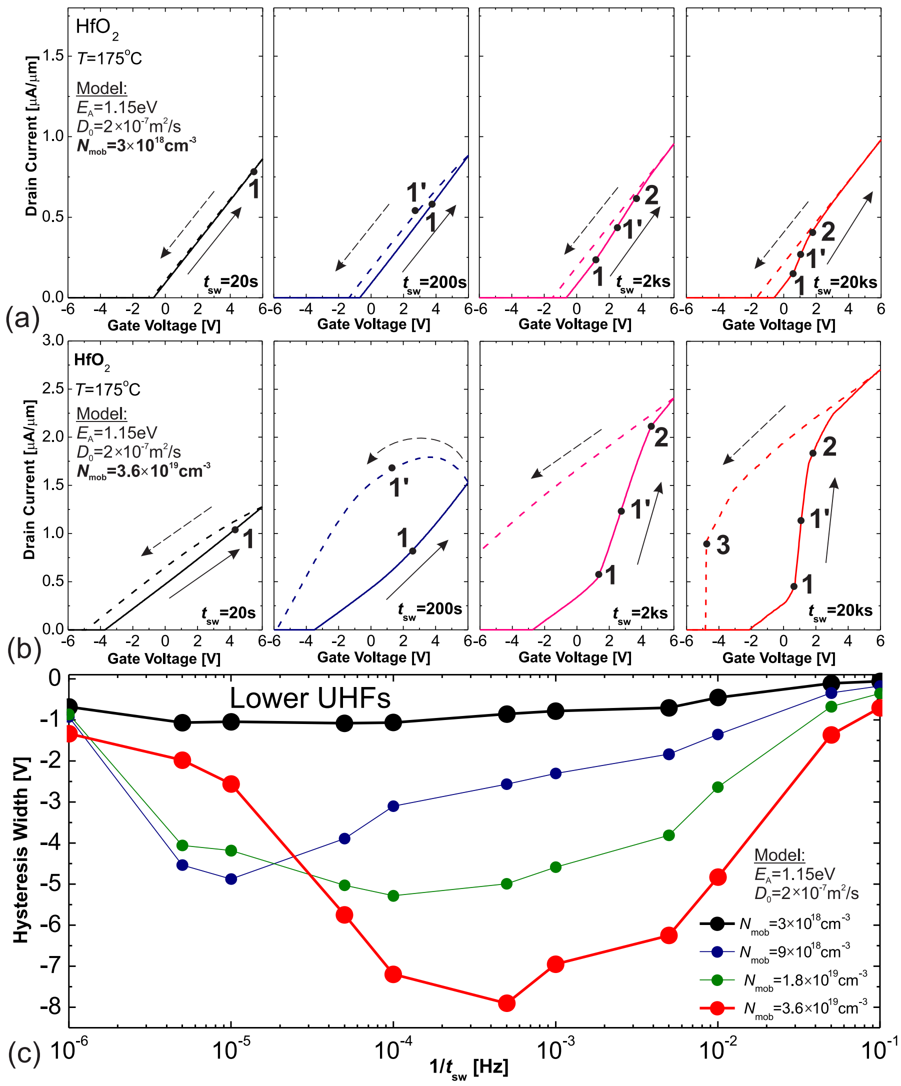

Figure S4: **Modeling results for different concentrations of mobile ions.** (a) Double sweep I_D-V_G characteristics of the MoS_2/HfO_2 FET simulated with our compact model considering reduced $N_{mob} = 3 \times 10^{18}\,cm^{-3}$. No TNDR effect is observed while an increase in I_{ON} is minor. (b) The related results obtained using $N_{mob} = 3.6 \times 10^{19}\,cm^{-3}$. Strong TNDR effect is present at moderate t_{sw}. (c) Lower UHFs extracted from the simulation results obtained using different N_{mob}. If there are too many mobile charges in the oxide, the maximum of CCW hysteresis is larger and appears at faster sweeps.

is larger, while the corresponding maximum is more distinct and appears at faster sweep frequencies.

In Fig.S5 we show that the pre-factor of the diffusion coefficient D_0 also affects the dynamics of CCW hysteresis considerably. If D_0 is made smaller, the ions obviously become

slower and need more time to cross the oxide thickness. This appears in the lower UHFs as a parallel shift of the maximum to slower frequencies. At the same time, D_0 does not affect the shape of the maximum, which is different from the impact of N_{mob} illustrated in Fig.S4c.

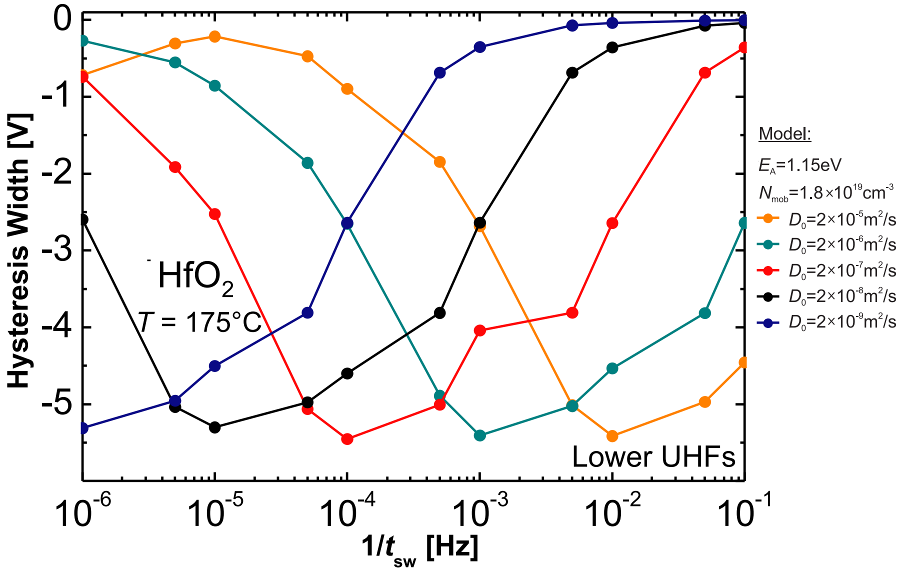

Figure S5: **Modeling results for different diffusion pre-factors.** Lower UHFs extracted from the simulation results for the MoS_2/HfO_2 FET obtained using different D_0. Smaller diffusion pre-factor obviously shifts the maximum of CCW hysteresis to slower sweep frequencies.

Equivalent sets of parameters in the compact model

The dynamics of mobile ions in HfO_2 will be mostly determined by N_{mob}, E_{A} and D_0 which are not known precisely. While our analysis qualitatively reproduces experimental findings using the Set 1 with $N_{\mathrm{mob}} = 3 \times 10^{19}\,\mathrm{cm}^{-3}$, $E_{\mathrm{A}} = 1.15\,\mathrm{eV}$ and $D_0 = 2 \times 10^{-7}\,\mathrm{m}^2/\mathrm{s}$, it is obvious that because of several degrees of freedom the compact model can produce identical hysteresis dynamics also using alternative combinations of parameters. Few examples that would be still physically feasible are listed in Fig.S6a and indeed produce the same hysteresis dynamics as illustrated by the lower UHFs provided in Fig.S6b.

We see that the model is very sensitive to E_{A} and even a minor increase of the activation

Set	E_A [eV]	D_0 [m^2/s]	N_{mob} [cm^{-3}]
**1**	**1.15**	**2.0×10^{-7}**	**1.8×10^{19}**
2	1.20	7.3×10^{-7}	1.8×10^{19}
3	1.05	1.64×10^{-8}	1.8×10^{19}

(a)

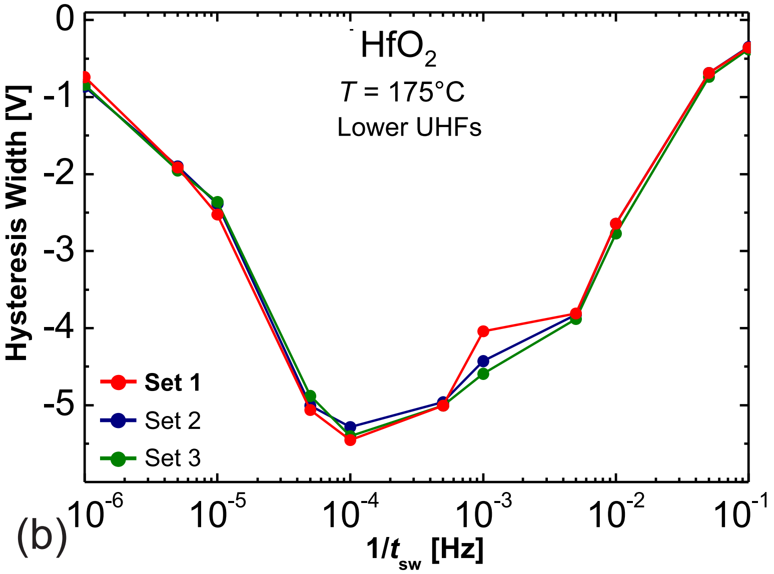

(b)

Figure S6: **Modeling results for equivalent sets of parameters.** (a) Examples of physically feasible equivalent sets of the key parameters of mobile charges in HfO_2 that would result in identical hysteresis dynamics according to the model. (b) The lower UHFs extracted from the I_D-V_G curves simulated using these 3 parameter sets that indeed look the same.

barrier would need a considerably larger D_0 (Set 2). Alternatively, a minor decay in E_A would require a much smaller D_0 to produce the same CCW hysteresis (Set 3). Therefore, we can conclude that considerable deviation of E_A from the range 1.05-1.25 eV that should be typical for oxygen vacancies in HfO_2 would likely result in non-physical values of N_{mob} and D_0.

Full mapping results for the sweep range dependence of the CCW hysteresis in MoS_2/HfO_2 FETs

In Fig.S7 we provide the full set of I_D-V_G characteristics and the corresponding mapping results measured for our MoS_2/HfO_2 FET at $T = 175°C$ using different V_G sweep ranges. It is obvious that for narrower sweep ranges TNDR effects and CCW hysteresis maximum are observed at considerably slower sweeps. This is because mobile charges in HfO_2 need more time to reach the channel side if V_{Gmax} is smaller. As a result, purely CW hysteresis is present for faster sweep frequencies in the case of -6 to 2 V and -6 to 4 V sweep ranges, thereby indicating that mobile charges are still unable to fully compensate charge trapping. Remarkably, with the full mapping results we can clearly identify and separate the frequency ranges with the purely CW hysteresis, CW/CCW switching and purely CCW hysteresis.

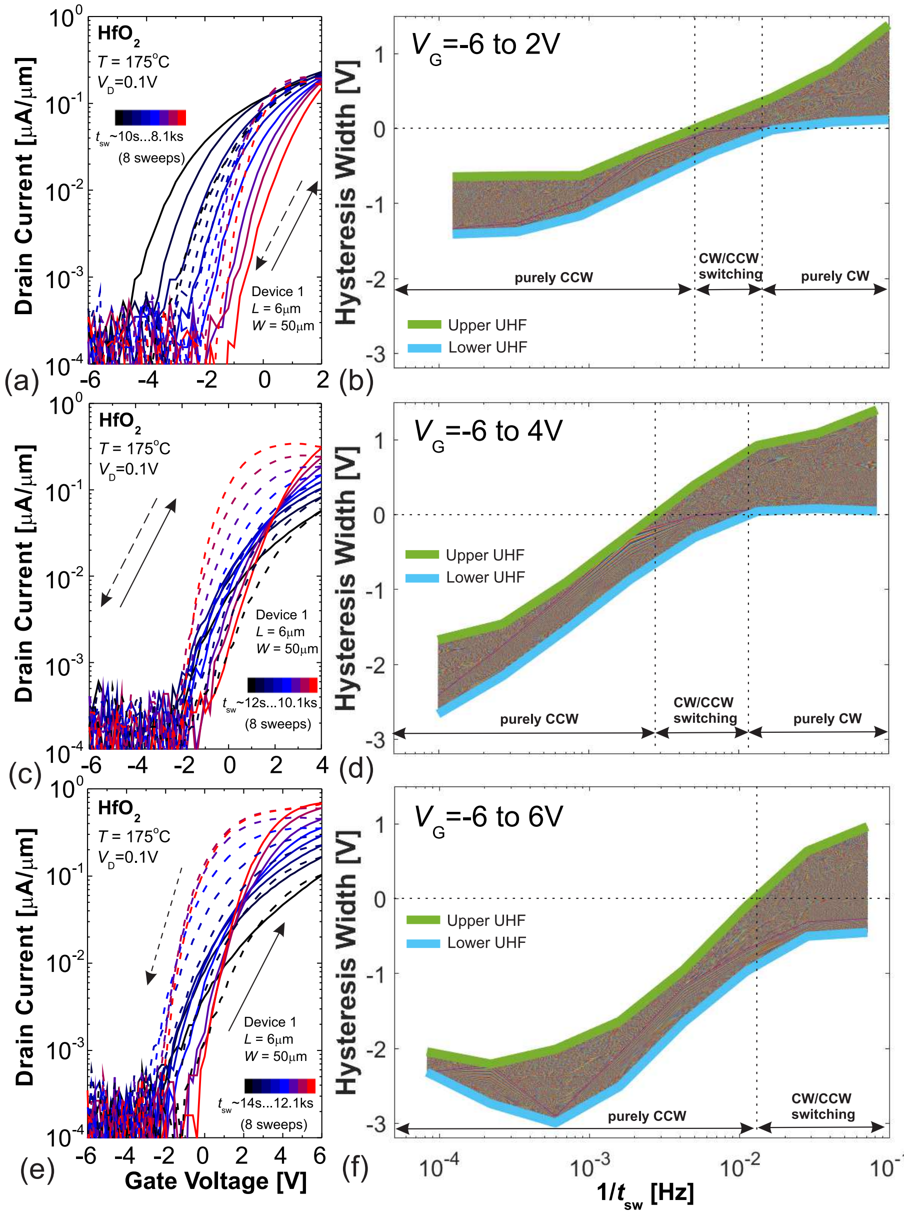

Figure S7: **Full mapping results for different sweep ranges.** Double sweep I_D-V_G characteristics of our MoS_2/HfO_2 FET measured at $T = 175$°C using 8 subsequent sweeps with t_{sw} up to 8.1, 10.1 or 12.1 ks and full hysteresis mapping results for the sweep ranges of -6 to 2 V (a,b), -6 to 4 V (c,d) and -6 to 6 V (e,f). Frequency ranges with different hysteresis dynamics can be clearly separated based on the signs of the upper and lower UHFs.

Sweep range and temperature dependence of the CCW hysteresis in MoS_2/HfO_2 FETs: compact model

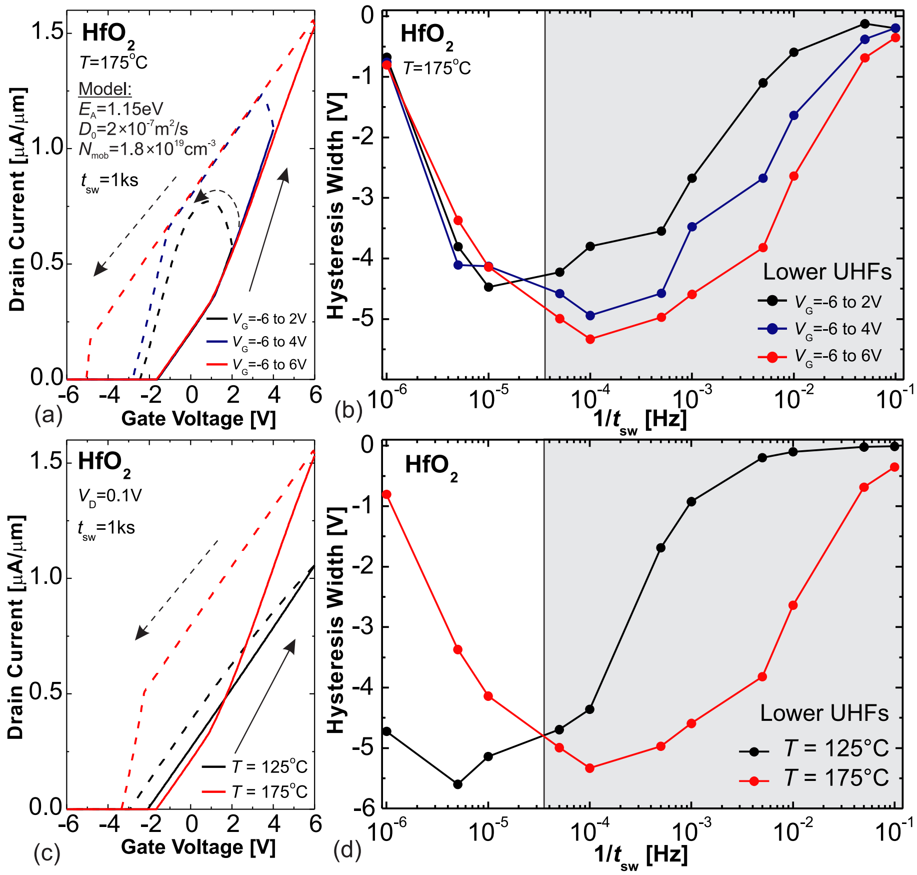

Figure S8: **Modeling results for the sweep range and temperature dependence of the counterclockwise hysteresis.** (a) Double sweep I_D-V_G characteristics of the MoS_2/HfO_2 FET simulated using our compact model while assuming different V_G sweep ranges, t_{sw} = 1 ks and T = 175°C. Just like in the experiments, for -6 to 2 V we still observe TNDR effect but for -6 to 6 V localization of the CCW starts. (b) The corresponding lower UHFs also confirm that acceleration of mobile charges with more positive V_{Gmax} is well described by the model. (c) Double sweep I_D-V_G characteristics of the MoS_2/HfO_2 FET simulated with the same model parameters for T = 125°C and 175°C assuming t_{sw} = 1 ks. At T = 175°C the CCW hysteresis is obviously larger. (d) The corresponding lower UHFs confirm that at higher temperature the CCW hysteresis maximum shifts to faster sweep frequencies.

In Fig.S8a,b we provide the results obtained using our compact model for different V_G sweep ranges. Faster change of the CCW hysteresis from the TNDR to localized behavior

for wider sweep ranges observed in the experiments (e.g. Fig.S7) is nicely confirmed by the I_D-V_G characteristics shown in Fig.S8a. The corresponding lower UHFs (Fig.S8b) show that for narrower sweep range the maximum of the CCW hysteresis shifts to slower sweep frequencies, which also goes in line with our experimental findings. Finally, in Fig.S8c,d we show the modeling results for T = 125°C and 175°C. They nicely confirm that the CCW hysteresis maximum shifts to faster sweep frequencies due to thermal activation of mobile charges which we also observe in our experiments.

Reproducibility of TNDR behavior in MoS_2/HfO_2 FETs

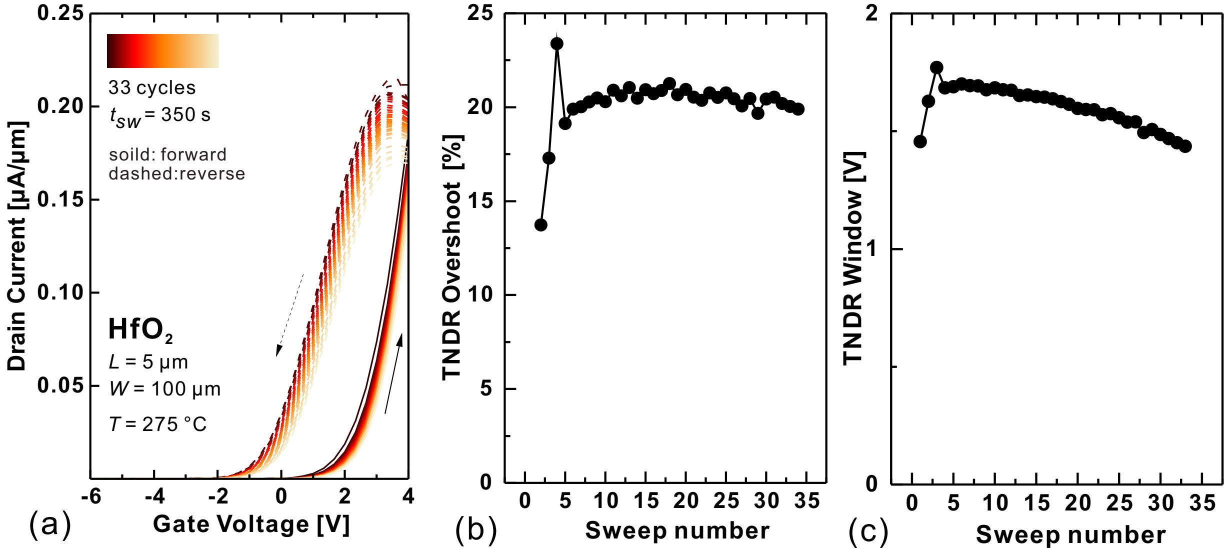

Figure S9: **Multi-sweep measurement results.** Cycle-to-cycle reproducibility of the TNDR behavior in MoS_2/HfO_2 FETs measured at 275°C with identical sweep time $t_{sw} \approx$ 350 s and V_G range of −6 to 4 V for all 33 cycles. (a) Consecutive double-sweep I_D-V_G curves, with color gradient indicating the sweep number. TNDR overshoot (b) and TNDR window (c) as a function of sweep number.

To confirm that the observed TNDR dynamics arise from the intrinsic migration of mobile oxygen vacancies rather than progressive degradation of the dielectric layer, we carried out 33 consecutive double I_D-V_G sweeps on the MoS_2/HfO_2 FET at 275°C (V_G range: −6 to 4 V, $t_{sw} \approx 350$ s). As shown in Fig.S9a, the I_D-V_G curves and TNDR features remain reasonably reproducible across all 33 cycles, with no observable dielectric degradation. To quantify the

long-term stability, we extracted two key TNDR metrics: the TNDR overshoot (defined as the maximum relative drain current increase during the reverse sweep, plotted in Fig.S9b) and the TNDR window (defined as the gate voltage span of the TNDR region, plotted in Fig.S9c). Both metrics are stable throughout the repeated sweeps, with some initial fluctuations and possible minor decay due to trapping of some mobile charges near the interfaces as the number of sweeps increases.

Full mapping results for hysteresis in MoS_2/Al_2O_3 FETs up to 275°C

In Fig.S10 we provide the full set of I_D-V_G characteristics and the corresponding mapping results measured for our MoS_2/Al_2O_3 FET at $T = 175$°C, $T = 225$°C, $T = 250$°C and $T = 275$°C. These results show that up to $T = 250$°C we are dealing with the purely CW hysteresis which appears as a bell-shape maximum of the upper UHFs that shifts to faster frequencies due to thermal activation. This behavior originates from charge trapping by oxide traps in Al_2O_3 that also causes a typical transformation of the shape of I_D-V_G curves with current decay for slower sweeps. At $T = 250$°C a certain compensation of the CW hysteresis at slow sweeps may be present, and finally at $T = 275$°C the slow sweep hysteresis is purely CCW. This reveals activation of mobile oxygen vacancies that comes together with the current increase for slow sweeps.

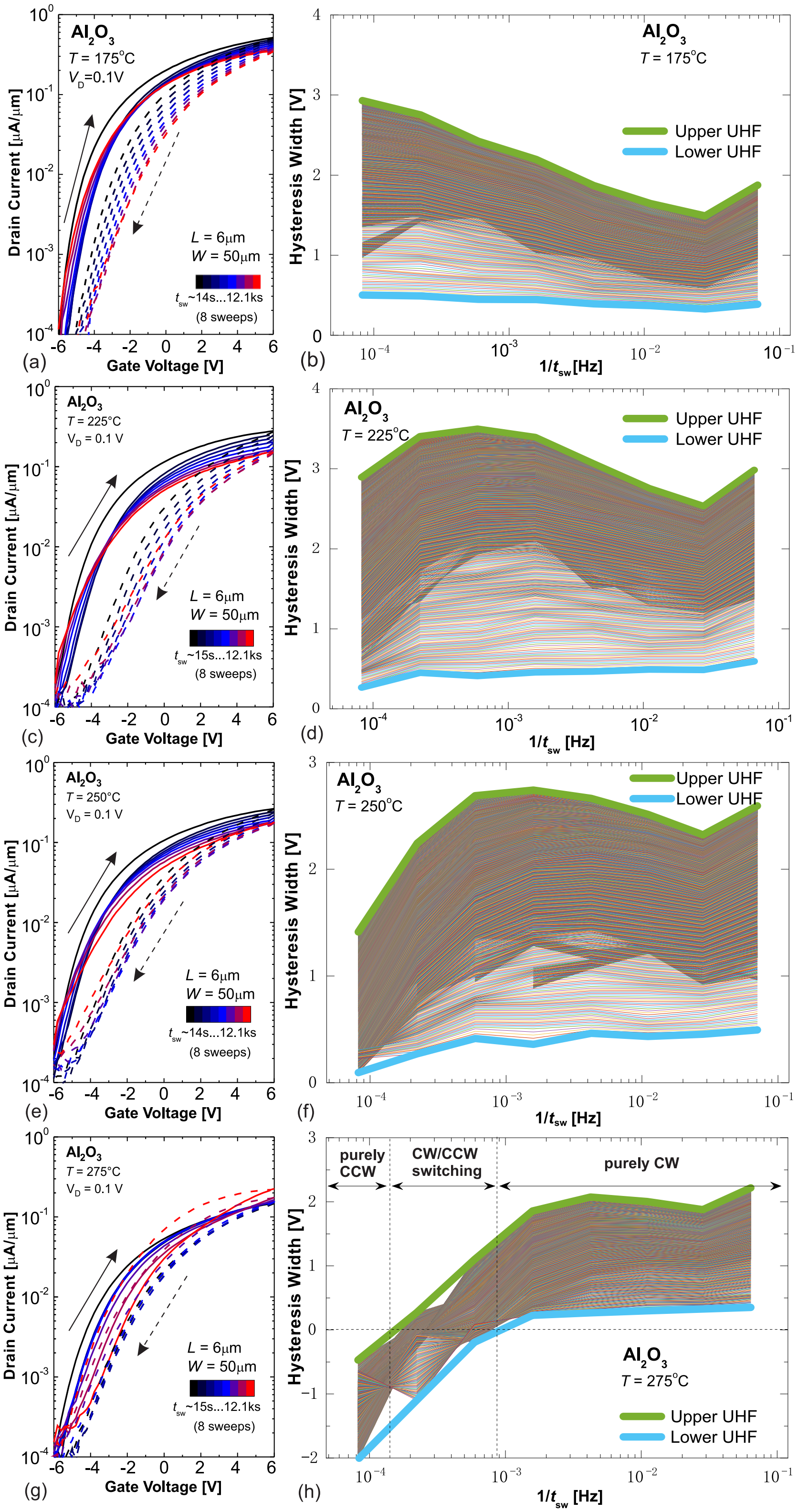

Figure S10: **High-temperature hysteresis in MoS_2/Al_2O_3 FETs via full mapping.** Double sweep I_D-V_G characteristics of our MoS_2/Al_2O_3 FET measured using 8 subsequent sweeps with t_{sw} up to 12.1 ks and full hysteresis mapping results for T = 175°C (a,b); T = 225°C (c,d); T = 250°C (e,f); T = 275°C (g,h).

Measurements of the gate leakage currents at high temperatures and slow sweeps

To verify the dielectric integrity and rule out the impact of gate leakage on the observed hysteresis dynamics, we measured the gate leakage currents (I_G) on both MoS_2/HfO_2 and MoS_2/Al_2O_3 FETs at representative high temperatures (175°C for HfO_2, 275°C for Al_2O_3) while doing hysteresis sweeps. As shown in Fig.S11, the gate leakage current remains at the noise level of Keithley 4200A. This confirms that our devices maintain excellent dielectric integrity without breakdown or excessive leakage at elevated temperatures.

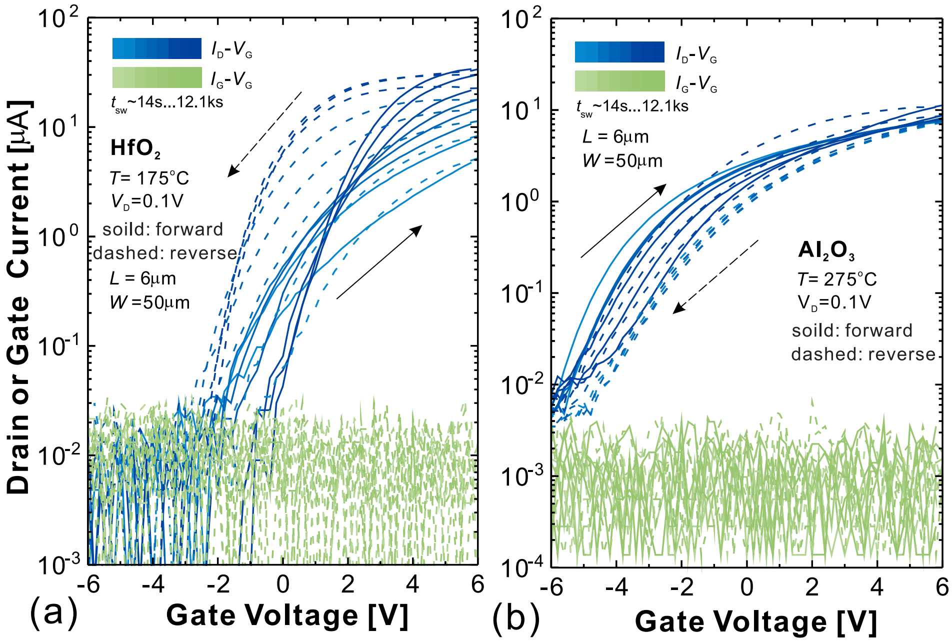

Figure S11: **Double sweep I_D–V_G curves and the corresponding gate leakage currents.** (a) Measured for MoS_2/HfO_2 at 175°C, (b) for MoS_2/Al_2O_3 at 275°C under vacuum and dark conditions. The gate leakage currents remain small across the entire V_G sweep range also for the slowest sweep times, confirming excellent dielectric integrity at elevated temperatures.

Qualitative modeling of $I_D(t)$ traces for MoS_2/HfO_2 FETs

In Fig.S12 we show the $I_D(t)$ dependences for a MoS_2/HfO_2 FET at $T = 175$°C obtained using our compact model for mobile charges with the parameters similar to the ones used for hysteresis. The key trends are the same as in our experiments. Namely, the current increases versus time as positive mobile charges come closer to the channel side of HfO_2. At a certain time saturation of I_D takes place when all of them reach their equilibrium positions. This saturation happens faster if V_{GS} is more positive. However, we note that the initial parts of these traces depend on the starting position of ions input into the simulations, or in fact in their starting distribution in the oxide. Here we consider an averaged value of $0.75 d_{ox}$.

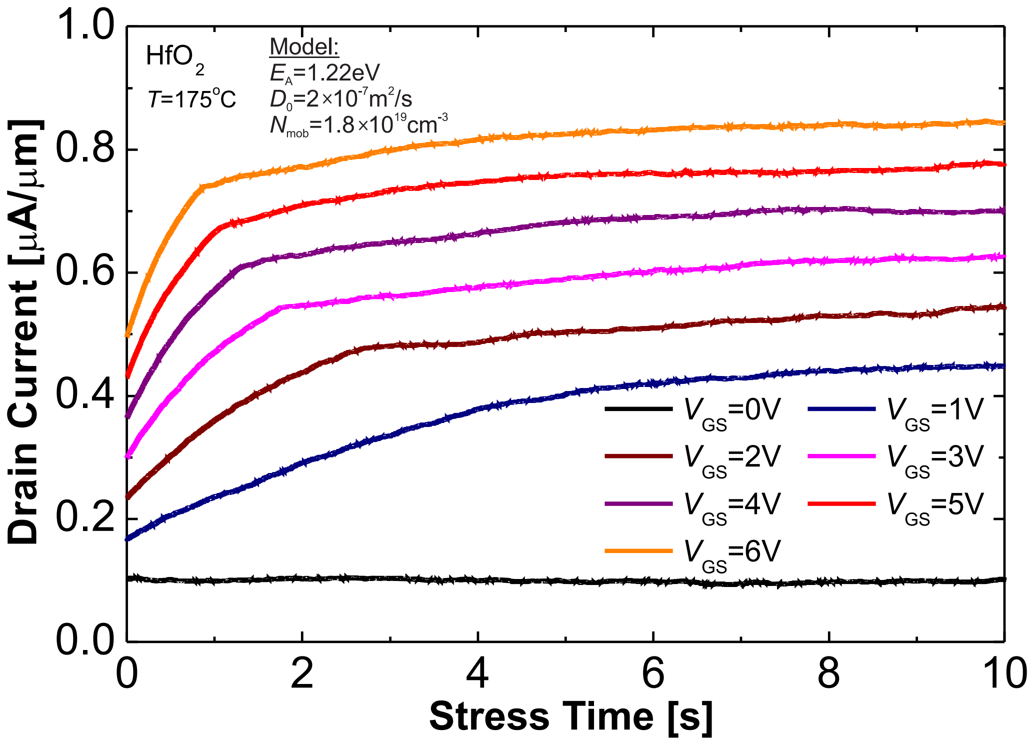

Figure S12: **Modeling results for constant bias stress.** Simulated $I_D(t)$ dependences for a MoS_2/HfO_2 FET at $T = 175$°C. Larger V_{GS} makes saturation of I_D faster since mobile charges need less time to cross the oxide.
