# Supplementary material for: Mobile charges in MoS2/high-k oxide transistors: from abnormal instabilities to transient negative differential resistance
Source: Commun Eng. 2026 Jul 2;5:120. doi: 10.1038/s44172-026-00716-2 (PMC13333013; doi:10.1038/s44172-026-00716-2)
Supplement: Supplementary file 2 — Transparent Peer Review File [file 44172_2026_716_MOESM2_ESM.pdf]

nature portfolio

Peer Review File

# Mobile charges in MoS2/high-k oxide transistors: from abnormal instabilities to transient negative differential resistance

Corresponding Author: Professor Yury Illarionov

**This file contains all reviewer reports in order by version, followed by all author rebuttals in order by version.**

Version 0:

Reviewer comments:

Reviewer #1

(Remarks to the Author)
The manuscript presents an extensive experimental study of time- and temperature-dependent hysteresis in $MoS_2$/high-k FETs and proposes a physical interpretation distinguishing trap-controlled CW hysteresis from mobile-charge-driven CCW dynamics. The comparison between $HfO_2$ and $Al_2O_3$ under identical fabrication conditions is valuable, and the use of full hysteresis mapping (upper/lower UHFs) helps avoid ambiguity associated with single-point constant-current extraction. The central observation (transition from trap-dominated CW hysteresis to vacancy-drift-driven CCW dynamics and memory-like NDR in $HfO_2$) is interesting and potentially impactful. However, the current revision still contains some issues. I therefore recommend acceptance after revision, provided the authors address the required clarifications and tighten the methodological description and figure/text consistency.The following points are intended to be constructive and to help improve clarity, reproducibility, and the strength of the mechanistic claims.
Major points
1. Temperature range inconsistency.
The manuscript states that measurements are performed up to 275 °C (main text; Fig. 6; and SI data such as Fig. S9). However, the Methods section ("Electrical characterization") states that measurements were performed from 25 °C to 175 °C. Please correct this inconsistency and ensure the temperature range is stated consistently throughout (main text, Methods, and SI).
2. Attribution of CCW/NDR to oxygen-vacancy drift: strengthen and bound the claim.
The interpretation that CCW hysteresis/NDR and negative Vth shifts in $HfO_2$ devices arise from drift of positively charged oxygen vacancies (V_O^+ / V_O^{2+}) is plausible and supported by thermal activation, sweep-range dependence, and bias-stress trends. Nevertheless, the manuscript would benefit from (i) a clearer discussion of alternative mobile species/processes (e.g., hydrogen-related ions, interfacial redox, charge injection into border traps) and (ii) additional justification for why oxygen vacancies are the dominant candidates under the present processing/measurement conditions. If direct chemical verification is not feasible, please frame the conclusion as 'most consistent with' rather than fully proven, and support it with targeted literature specific to oxygen vacancy mobility in ALD $HfO_2$/$Al_2O_3$.
3. Gate leakage and dielectric integrity at high temperature.
Given high-temperature operation and the interpretation involving oxide defects/mobile charges, it would strengthen the work to include representative gate-leakage (I_G–V_G) curves at room temperature and at key elevated temperatures (e.g., 175 °C for $HfO_2$ and 275 °C for $Al_2O_3$). This helps confirm that the observed effects are not influenced by significant leakage or oxide breakdown.
4. Universal hysteresis mapping method: ensure self-containment.
Because the universal mapping approach is central and the cited reference is an arXiv preprint, please ensure the main text contains enough methodological detail for readers to reproduce the extraction (definition of ΔV_H, how constant-current points are sampled, criteria for upper/lower UHFs, and sign convention for CW vs CCW). Consider adding a short 'How UHFs are extracted' paragraph in Methods.
5. Compact model: parameter definitions, sign conventions, and identifiability.
To avoid confusion in the modeling:
• Clearly define the sign convention for ΔVth and for hysteresis width in both main text and SI.
• In the SI, the interface state density Dit is introduced with a negative value to emulate acceptor-type charges. Since densities are non-negative, please re-express this as a positive Dit with an explicit sign for the associated charge, or define

a separate fixed charge term.
• When reporting ‘best agreement’ parameters (EA, N_mob, D0), emphasize that these are qualitative/representative and bounded, not unique fits.
6. Editorial quality and figure/text consistency.
Please correct typos and language issues (e.g., the section heading “Investigated devices and mesurement technique”, and other recurring misspellings/awkward phrasing). Also verify that all figure captions and in-text references are fully consistent (panel labels, temperature, sweep range, and which curves are ‘upper’ vs ‘lower’).
Minor points and suggestions
• $MoS_2$ monolayer confirmation: Consider adding a brief characterization summary (e.g., representative Raman/PL and/or AFM thickness) in the main text or SI to confirm monolayer nature and uniformity, since stability can depend on defects/contamination introduced during wet transfer.
• NDR metric: Please provide an explicit definition/equation for the ‘NDR magnitude’ used in Fig. 5d and clarify whether it is normalized or absolute.
• Terminology: Use ‘clockwise (CW)’ and ‘counter-clockwise (CCW)’ with a consistent sign convention across all figures (main and SI).

Reviewer #2

(Remarks to the Author)
The manuscript presents an interesting study on hysteresis dynamics in $MoS_2/HfO_2$ and $MoS_2/Al_2O_3$ FETs and proposes the use of $HfO_2$ as an intrinsic memory layer based on CCW hysteresis and NDR behavior. While the results are intriguing, several points require clarification.

1 If $HfO_2$ is proposed as an intrinsic memory layer, additional discussion on retention and endurance characteristics would be necessary to support this claim. Without such data, it is difficult to assess the practical feasibility of the proposed memory functionality.

2. The CCW hysteresis and NDR behavior are attributed primarily to mobile oxygen vacancy drift in the oxide. It would be helpful to further clarify whether this phenomenon is inherently linked to the 2D nature of the $MoS_2$ channel or whether it represents a more general high-k oxide effect that could also appear in conventional Si MOSFETs. A clearer discussion of the role of the $MoS_2$ channel in amplifying or enabling this behavior would strengthen the manuscript.

3. While the mobile vacancy drift model qualitatively explains the observations, additional justification is needed to rule out alternative mechanisms such as deep trap reconfiguration or defect generation at elevated temperatures.

4. Since the oxide thickness is fixed at 20 nm throughout the study, it is unclear whether the proposed drift mechanism can be generalized without thickness-dependent analysis. Discussion of thickness scaling effects would improve the robustness of the conclusions.

5. It would also be useful to comment on whether similar CCW dynamics have been observed in other high-k dielectrics, to clarify whether the phenomenon is specific to $HfO_2$.

6. Clarification is needed regarding the reproducibility of the NDR behavior over repeated sweeps. It is important to distinguish whether this effect represents a stable functional mechanism or the onset of progressive dielectric degradation.

Overall, the study is interesting, but addressing the points above would significantly strengthen the physical interpretation and practical implications of the work.

Reviewer #3

(Remarks to the Author)
Dear authors,

The manuscript titled “Mobile charges in molybdenum disulfide/high-k oxide transistors: from abnormal instabilities to memory-like dynamics” presents a detailed characterization of the hysteretic behavior in MoS2/HfO2 and MoS2/Al2O3 field-effect transistors. The authors successfully highlight the transition from CW hysteresis at room temperature to CCW hysteresis with NDR at elevated temperatures. They propose a compact model attributing these effects to the drift of mobile oxygen vacancies within the oxide.
While the physical mechanisms behind the hysteresis are discussed in depth, the memory-like dynamics mentioned in the title are not sufficiently explored and convincingly demonstrated. The current focus remains heavily on the physical explanation of high-temperature counter-clockwise hysteresis rather than on practical memory applications or performance metrics. Therefore, while the work is technically interesting, it requires major revisions to better align the results with the stated claims before it can be considered for publication in Communications Engineering.

1. MoS2 hysteresis is frequently associated with ambient adsorbates. While this study was conducted in vacuum, this detail is missing from the abstract and the specific vacuum level is not reported in the results. The authors should clarify these experimental conditions to ensure data reproducibility.
2. The authors claim the MoS2 under study is a monolayer, yet no AFM profile, flake image, or material characterization is

provided to support this. Additionally, the optical image in Figure 1(b) should be shown at a higher magnification to clearly resolve the flake.
3. The output curves in the Figure 1(c) inset indicate Schottky behavior. While the compact model assumes a Schottky barrier of 0.2 eV, the manuscript does not specify if this value was derived from standard Richardson analysis or band-alignment observations. Since non-homogeneous Schottky barriers can significantly enhance hysteresis in TMD-based FETs (see https://doi.org/10.1002/aelm.202500327), the authors should account for this effect in their discussion.
4. The curves in Figure 2 are presented in a confusing manner. Specifically, the dashed line representing the other branch of the transfer curve is not defined in the text or the caption. Additionally, the band-alignment diagram in Figure 2(e) lacks labels for the energy levels, such as Ec and Ev. The authors should clarify these graphical elements to improve the readability of the data.
5. The sentence “Furthermore, DVh starts increasing at smaller sweep frequencies as these …” at page 8 lacks literature reference.
6. The increase in the ON current observed with increasing sweep times should also be considered in relation to potential temperature increments resulting from prolonged measurement durations. The authors should discuss whether self-heating contributes to this behavior.
7. The authors explain that the CCW hysteresis and the NDR effect is also present in FETs at 125°C and with slower time of sweep and relate this possibility to devices with more vacancies in the oxide. This possibility makes more difficult the reproducibility and the control on the behavior of the device. The authors should discuss some engineering of defects method to clarify in which sense this type of device should be considered for memory-like dynamics.
8. Several parameters in the compact model appear arbitrary. The effective mobility of 10 cm2 V-1 s-1 for MoS2 should be justified by specifying if it was extracted via transfer curves, the Y-function method, or output curves. The assumed bandgap of 2.53 eV is significantly higher than the widely reported value of approximately 1.8 eV (https://doi.org/10.3389/fchem.2021.700250). The subthreshold swing of 280 mV/decade should also be verified against experimental data. Notably, the temperature dependence of the subthreshold swing could provide an estimation of interface state density and its role in the observed hysteresis (see again https://doi.org/10.1002/aelm.202500327). Finally, the selection of the Nmob value must be clearly explained to establish the reliability of the model.
9. The device stability under bias stress is well presented; however, the memory effect, despite being a central theme of the title, remains unclear. Essential metrics for a functional memory device, such as write/read/erase operations, endurance, and retention, are not reported. Consequently, the work currently functions as a characterization of hysteresis rather than a demonstration of a memory device. The authors should discuss these limitations and consider revising the title to better align with the data actually presented in the manuscript.

Reviewer #4

(Remarks to the Author)
I co-reviewed this manuscript with one of the reviewers who provided the listed reports. This is part of the Communications Engineering initiative to facilitate training in peer review and to provide appropriate recognition for Early Career Researchers who co-review manuscripts.

Version 1:

Reviewer comments:

Reviewer #1

(Remarks to the Author)
The authors have addressed this reviewer’s comments satisfactorily. I recommend publication of the article in its present form.

Reviewer #2

(Remarks to the Author)
The authors have satisfactorily addressed the reviewer’s comments and significantly improved the clarity and technical strength of the manuscript.

Reviewer #3

(Remarks to the Author)
Dear authors,
I acknowledge the efforts made to improve the scientific rigor, readability, and reproducibility of your work. I recommend the revised version of the paper for publication in Communications Engineering.

Reviewer #4

(Remarks to the Author)
I co-reviewed this manuscript with one of the reviewers who provided the listed reports. This is part of the Communications Engineering initiative to facilitate training in peer review and to provide appropriate recognition for Early Career Researchers who co-review manuscripts.

**Open Access** This Peer Review File is licensed under a Creative Commons Attribution 4.0 International License, which permits use, sharing, adaptation, distribution and reproduction in any medium or format, as long as you give appropriate credit to the original author(s) and the source, provide a link to the Creative Commons license, and indicate if changes were made.
In cases where reviewers are anonymous, credit should be given to 'Anonymous Referee' and the source.
The images or other third party material in this Peer Review File are included in the article's Creative Commons license, unless indicated otherwise in a credit line to the material. If material is not included in the article's Creative Commons license and your intended use is not permitted by statutory regulation or exceeds the permitted use, you will need to obtain permission directly from the copyright holder.
To view a copy of this license, visit https://creativecommons.org/licenses/by/4.0/

## Rebuttal letter

*Manuscript title (updated):*

**Mobile charges in $MoS_2$/high-k oxide transistors: from abnormal instabilities to transient negative differential resistance**

*Authors*: Shaokai Zhou, Haihui Cai, Yehao Wu, Yufeng Min, Renchen Yuan,P Yezhu Lv, Jianming Huang, Yuanyuan Shi, and Yury Yuryevich Illarionov*,

*Journal*: Communications Engineering

Manuscript number: COMMSENG-25-0997-T

We thank all reviewers for their valuable comments. We have revised the manuscript carefully according to the reviewers' suggestions and editor's detailed instructions. All changes are highlighted in red in the revised manuscript and SI. Below is our point-by-point response.

## Reviewer 1

**Comment 0:**

*The manuscript presents an extensive experimental study of time- and temperature-dependent hysteresis in MoS2/high-k FETs and proposes a physical interpretation distinguishing trap-controlled CW hysteresis from mobile-charge-driven CCW dynamics. The comparison between HfO2 and Al2O3 under identical fabrication conditions is valuable, and the use of full hysteresis mapping (upper/lower UHFs) helps avoid ambiguity associated with single-point constant-current extraction. The central observation (transition from trap-dominated CW hysteresis to vacancy-drift-driven CCW dynamics and memory-like NDR in HfO2) is interesting and potentially impactful. However, the current revision still contains some issues. I therefore recommend acceptance after revision, provided the authors address the required clarifications and tighten the methodological description and figure/text consistency.The following points are intended to be constructive and to help improve clarity, reproducibility, and the strength of the mechanistic claims.*

**Answer:** We thank the reviewer for positive evaluation of our manuscript, the detailed point-by-point replies are provided below.

**Comment 1:**

*Temperature range inconsistency. The manuscript states that measurements are performed up to 275 °C (main text; Fig. 6; and SI data such as Fig. S9). However, the Methods section ("Electrical characterization") states that measurements were performed from 25 °C to 175 °C. Please correct this inconsistency and ensure the temperature range is stated consistently throughout (main text, Methods, and SI).*

**Answer:** We thank the reviewer for noting this. We have unified the temperature range to 25 °C – 275 °C throughout the main text, Methods, and SI. The caption of Fig. 6 has been corrected accordingly.

**Comment 2:**

*Attribution of CCW/NDR to oxygen-vacancy drift: strengthen and bound the claim. The interpretation that CCW hysteresis/NDR and negative Vth shifts in HfO2 devices arise from drift of positively charged oxygen vacancies (VO +/VO^(2+}) is plausible and supported by thermal activation, sweep-range dependence, and bias-stress trends. Nevertheless, the manuscript would benefit from (i) a clearer discussion of alternative mobile species/processes*

*(e.g., hydrogen-related ions, interfacial redox, charge injection into border traps) and (ii) additional justification for why oxygen vacancies are the dominant candidates under the present processing/measurement conditions. If direct chemical verification is not feasible, please frame the conclusion as 'most consistent with' rather than fully proven, and support it with targeted literature specific to oxygen vacancy mobility in ALD $HfO_2/Al_2O_3$.*

**Answer:** We thank the Reviewer for this important comment. First, charge trapping by border traps and interfacial redox (e.g. due to adsorbates) would result in a CW hysteresis contribution. The former is what we observe at room temperature, and the latter can be neglected considering measurements in vacuum at elevated temperatures which desorbs typical redox-active species like $H_2O/O_2$. The CCW hysteresis that we observe is related to mobile charges in oxides, as confirmed by our previous analysis (e.g. T. Knobloch et al, IEEE IEDM 2023) and the extended model that also explains TNDR effects that we use in this work. Indeed, there can be alternative mobile charges. For instance, $H^+$ is ubiquitous in thin film FETs as it is present in amorphous oxide semiconductors. However, these are much faster mobile charges that produce the CCW hysteresis already at room temperature (e.g. S. Wahid et al, IEEE IEDM 2022). Other possible candidates are $K^+$ ions which are slower and have been shown to get activated only at 165°C (T. Knobloch et al, IEEE IEDM 2023), though without causing TNDR likely due to relatively small concentrations. However, in contrast to those $MoS_2/SiO_2$ FETs in which potassium salt (PTAS) was used as a catalyst for direct CVD growth of $MoS_2$ on $SiO_2$, in our case there is no source of $K^+$ as the $MoS_2$ films were transferred onto high-k oxides. In contrast, oxygen vacancies are known to be present in ALD-grown $HfO_2$ and $Al_2O_3$ (see e.g. Martínez-Puente et al., Mater. Sci. Eng. B 285, 115964, 2022) and should have sufficient concentrations to cause TNDR. For instance, usual CCW hysteresis due to $K^+$ can be well explained with $N_{mob}$ $=10^{18}$cm$^{-3}$ that is reasonable for contaminants coming from the device processing, and in this work we consider $N_{mob}$ $=3*10^{19}$cm$^{-3}$ to get TNDR which would make sense for intrinsic oxide defects like oxygen vacancies rather than for impurities. Furthermore, in amorphous oxides oxygen vacancies should be slower than in crystalline STO insulators where they cause analogous CCW dynamics at lower temperatures (see e.g. Sattari-Esfahlan et al., ACS Nano 19, 12288 2025). ***We have now added the discussion of alternative mobile ions and comparison of mobile charge concentrations with the previous work to pages 12,14 of the manuscript, while our interpretation is now framed as "most consistent with" oxygen vacancy drift rather than fully proven.***

**Comment 3:**

*Gate leakage and dielectric integrity at high temperature. Given high-temperature operation and the interpretation involving oxide defects/mobile charges, it would strengthen the work to include representative gate-leakage (IG-VG) curves at room temperature and at key elevated temperatures (e.g., 175 °C for HfO2 and 275 °C for Al2O3). This helps confirm that the observed effects are not influenced by significant leakage or oxide breakdown.*

**Answer:** We agree with the reviewer. We have performed gate leakage ($I_G$–$V_G$) measurements for both $MoS_2/HfO_2$ and $MoS_2/Al_2O_3$ FETs at the key elevated temperatures (175°C for $HfO_2$, 275°C for $Al_2O_3$). ***As shown in new Fig. S11,*** gate leakage currents remain at the measurement noise level across the entire $V_G$ sweep range, confirming that the devices maintain excellent dielectric integrity without breakdown or excessive leakage at high temperatures. Therefore, we can conclude that the the observed hysteresis dynamics are not affected by gate leakage. ***The corresponding comment is now added to the end of page 19 in the manuscript, along with new Fig.S11 and its discussion in page 19 of the SI.***

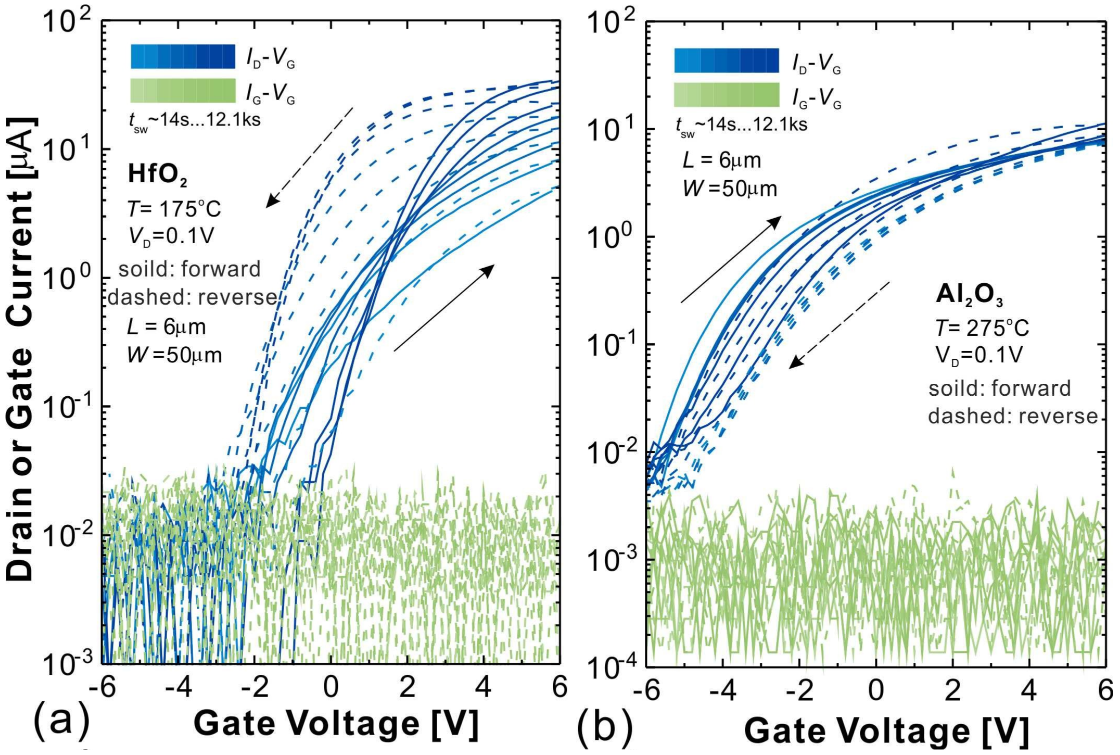


New Figure S11 showing negligible gate leakage currents at elevated temperatures for both types of our $MoS_2$ FETs.

**Comment 4:**

*Universal hysteresis mapping method: ensure self-containment. Because the universal mapping approach is central and the cited reference is an arXiv preprint, please ensure the main text contains enough methodological detail for readers to reproduce the extraction (definition of ΔVH, how constant-current points are sampled, criteria for upper/lower UHFs, and sign convention for CW vs CCW). Consider adding a short 'How UHFs are extracted' paragraph in Methods.*

**Answer:** We agree with the reviewer. ***The description of universal mapping method is now added to Methods (new subsection "Extraction of the hysteresis width", page 24).*** The corresponding paper which is already published is cited properly (see Y.Z. Lv et al, npj 2D Materials and Applications, 10, 35, 2026).

**Comment 5:**

*Compact model: parameter definitions, sign conventions, and identifiability. To avoid confusion in the modeling: Clearly define the sign convention for ΔVth and for hysteresis width in both main text and SI. In the SI, the interface state density Dit is introduced with a negative value to emulate acceptor-type charges. Since densities are non-negative, please re-express this as a positive Dit with an explicit sign for the associated charge, or define a separate fixed charge term. When reporting 'best agreement' parameters (EA, Nmob, D0), emphasize that these are qualitative/representative and bounded, not unique fits.*

**Answer:** We appreciate this comment of the reviewer and addressed it as follows:

- In the beginning of the new subsection of Methods (Extraction of the hysteresis width) ***it is now sated that the hysteresis with is positive for CW case and negative for CCW case***
- ***In the SI (step 6 of the model), we have removed the minus sign in $D_{it}$ to make the density a positive quantity***. Eq. (7) now includes an ***explicit negative sign to consider acceptor-type interface charges***, and Eq. (8) ***has been changed from a minus to a plus sign for the $D_{it}$ term*** to maintain the correct flat-band shift. We note that these changes do not affect the output results of our model.

As for $\Delta V_{th}$, next to the equation 6 in the SI it was already said that the minus sign is needed because we consider positive mobile charges which should create a negative $\Delta V_{th}$.

Regarding the qualitative parameters, in the text (page 15) it was already mentioned: "*This would obviously make it complicated to use precise fits of the experimental data for extraction of ion parameters. However, we can consider that $E_A$ values which we are using provide a reasonable estimate since any change for more than 0.2 eV would either result in a very different dynamics as compared to our experimental observations, or in non-physical values of $N_{mob}$ and $D_0$.*" ***This actually goes in line with the reviewer's comment and emphasizes that the parameters are bounded and representative, not unique fits.***

**Comment 6:**

*Editorial quality and figure/text consistency. Please correct typos and language issues (e.g., the section heading "Investigated devices and mesurement technique", and other recurring misspellings/awkward phrasing). Also verify that all figure captions and in-text references are fully consistent (panel labels, temperature, sweep range, and which curves are 'upper' vs 'lower').*

**Answer:** We thank the reviewer. We have carefully proofread the manuscript, corrected typos (e.g., "mesurement" → "measurement"), **and updated** the line styles (solid for forward, dashed for reverse) in Fig. 2–6 and added $E_C$/$E_V$ labels to the band diagram in Fig. 2e.

**Minor points and suggestions**

**Comment:** *MoS2 monolayer confirmation: Consider adding a brief characterization summary (e.g., representative Raman/PL and/or AFM thickness) in the main text or SI to confirm monolayer nature and uniformity, since stability can depend on defects/contamination introduced during wet transfer.*

**Answer:** We agree with the the reviewer. ***Raman spectrum confirming monolayer $MoS_2$ is now added as Fig.1b.***

**Comment:** *NDR metric: Please provide an explicit definition/equation for the 'NDR magnitude' used in Fig. 5d and clarify whether it is normalized or absolute.*

**Answer:** We agree with the the reviewer. ***We have replaced the old Fig. 5d with a new panel defining two metrics:*** (i) normalized TNDR overshoot $R$ = 100·($I_{peak}/I_{start}$ – 1), and (ii) TNDR window $\Delta V_{TNDR} \geq 0$ which is the voltage range over which reverse-sweep current remains above $I_{start}$ (i.e. the current at $V_{Gmax}$ during the forward sweep). ***The corresponding comments are added to the main text (pages 16-17) and in the new Fig.5d caption.***

**Comment:** Terminology: Use 'clockwise (CW)' and 'counter-clockwise (CCW)' with a consistent sign convention across all figures (main and SI).

**Answer:** For the CW hysteresis the width is positive, and for the CCW hysteresis it is negative. ***This is clearly stated in Methods.***

## Reviewer 2

**Comment 0:**

*The manuscript presents an interesting study on hysteresis dynamics in MoS2/HfO2 and MoS2/Al2O3 FETs and proposes the use of HfO2 as an intrinsic memory layer based on CCW hysteresis and NDR behavior. While the results are intriguing, several points require clarification.*

**Answer:** We thank the reviewer for positive evaluation of our manuscript, the detailed clarifications are provided below.

**Comment 1:**

*If HfO2 is proposed as an intrinsic memory layer, additional discussion on retention and endurance characteristics would be necessary to support this claim. Without such data, it is difficult to assess the practical feasibility of the proposed memory functionality.*

**Answer:** We thank the reviewer for this comment. ***We have now added new results for 33 consecutive double sweeps performed for $MoS_2/HfO_2$ FETs at 275 °C*** (see new Fig. S9 in the SI). The TNDR overshoot and window show reasonable stability over all cycles, confirming the intrinsic and reproducible nature of the observed dynamics. This stability, together with the absence of device failure, supports the potential of $HfO_2$-gated $MoS_2$ FETs for memory-like applications. However, in this work is the first study that reports TNDR dynamics and thus cannot answer all questions related to retention/endurance which are beyond the scope. ***Thus we have also adjusted the title of our manuscript to make it less memory-focused and highlight TNDR behavior instead.***

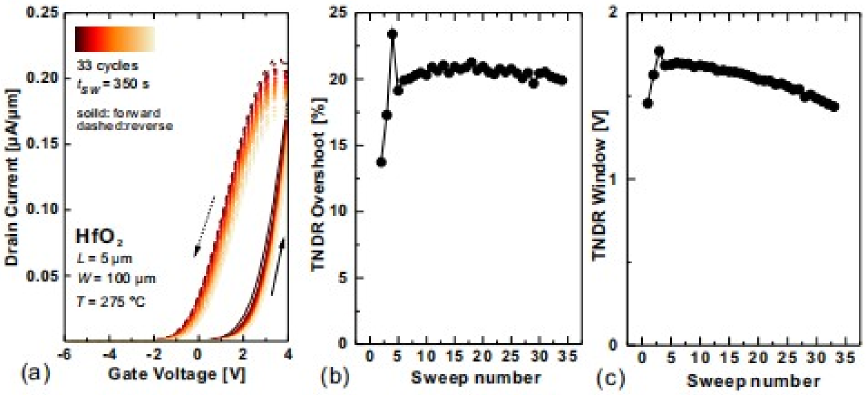


New Figure S9 showing reasonable reproducibility of TNDR behavior in $MoS_2/HfO_2$ FETs at 275°C with identical sweep time $t_{sw}$ ≈ 350s across 33 sweeps.

**Comment 2:**

*The CCW hysteresis and NDR behavior are attributed primarily to mobile oxygen vacancy drift in the oxide. It would be helpful to further clarify whether this phenomenon is inherently linked to the 2D nature of the MoS2 channel or whether it represents a more general high-k oxide effect that could also appear in conventional Si MOSFETs. A clearer discussion of the role of the MoS2 channel in amplifying or enabling this behavior would strengthen the manuscript.*

**Answer:** We thank the reviewer for this comment. Indeed, the CCW hysteresis and TNDR are related to the drift of mobile charges in oxides and not the 2D nature of the $MoS_2$ channel. In early silicon-based devices, the presence of mobile contaminants ($K^+$, $Na^+$, $Li^+$) also affected device reliability and performance (see e.g. G. Greeuw et al J. Appl. Phys. 56, 2218, 1984). Furthermore, the CCW hysteresis due to mobile charges was also observed in ITO/$HfO_2$ FETs (e.g. S. Wahid et al, IEEE IEDM 2022), as well as $MoS_2$ FETs gated with $SiO_2$ (T. Knobloch et al, IEEE IEDM 2023) and STO (M. Sattari-Esfahlan et al., ACS Nano 19, 12288 2025), see also our reply to the comment 2 of the reviewer 1. However, the presence of TNDR in addition to the CCW hysteresis, which is reported in this study for the first time, requires sizable concentration of mobile charges (see e.g. Fig.S4 in the SI) that is unlikely reachable for side contaminants coming from the oxide or channel growth processes. That is why we suggest that oxygen vacancies that are present in ALD-grown high-k oxides as intrinsic defects (see e.g. Martínez-Puente et al., Mater. Sci. Eng. B 285, 115964, 2022) should be the most likely candidates to cause TNDR behavior rather than simple CCW hysteresis that was observed previously. ***We have added the corresponding comment and additional citations to pages 12,14 of the manuscript.***

**Comment 3:**

*While the mobile vacancy drift model qualitatively explains the observations, additional justification is needed to rule out alternative mechanisms such as deep trap reconfiguration or defect generation at elevated temperatures.*

**Answer:** We thank the reviewer for this comment. Several observations rule out deep trap reconfiguration or irreversible defect generation: (i) the CCW hysteresis exhibits a distinct, bell-shaped maximum in $\Delta V_H(1/t_{sw})$ that shifts

with temperature (see e.g. Fig. 3 and Fig.S2). This is a characteristic of a thermally activated ion drift process (see T. Knobloch et al, IEEE IEDM 2023, M. Sattari-Esfahlan et al., ACS Nano 19, 12288 2025); (ii) the observed behavior is fully reversible and reproducible over multiple cycles with no increase in gate leakage or permanent drifts of $V_{th}$ (***see newly added Fig. S9 and Fig. S11***). Thus, mobile oxygen vacancy drift remains the most consistent explanation as highlighted by the results shown in our manuscript and SI.

**Comment 4:**

*Since the oxide thickness is fixed at 20 nm throughout the study, it is unclear whether the proposed drift mechanism can be generalized without thickness-dependent analysis. Discussion of thickness scaling effects would improve the robustness of the conclusions.*

**Answer:** We appreciate this comment of the reviewer. Actually in our compact model (see pages 6-7 in the SI) the total drift time that is required for mobile charges to cross the entire oxide thickness $\tau_{drift} = d_{ox}/v_{drift} = d^2_{ox}/(\mu|V_G|)$. As $\tau_{drift} \propto d^2_{ox}$, the use of thinner oxides would obviously make the ions to reach the $MoS_2/HfO_2$ faster. This will shift the maxima of CCW hysteresis, TNDR overshoot and TNDR window to faster sweep frequencies, though with possible decay in the effects due to trapping of ions at the interfaces. For instance, the use of smaller $d_{ox}$ is analogous to the use of larger $V_{Gmax}$ which is illustrated in Fig.5 and consistent with the same model. This suggests that the proposed mechanism of ion drift can be generalized based on the use of different $V_{Gmax}$, although tuning of TNDR effects with $d_{ox}$ to achieve faster operation could be indeed an interesting direction for future research. ***We have now added a brief comment on thickness scaling within the existing paragraph discussing the effect of $V_{Gmax}$, see pages 17-18 in the revised manuscript.***

**Comment 5:**

*It would also be useful to comment on whether similar CCW dynamics have been observed in other high-k dielectrics, to clarify whether the phenomenon is specific to HfO2.*

**Answer:** We agree with the reviewer. ***We have added the corresponding discussion to pages 12,14 in the manuscript, see also our reply to your***

***comment 2 and the comment 2 of the reviewer 1.*** For instance, similar CCW hysteresis was observed in FETs based on $MoS_2/SiO_2$ (Knobloch et al., *IEEE IEDM* 2023) and $MoS_2/SrTiO_3$ (Sattari-Esfahlan et al., *ACS Nano* 2025). However, those systems do not exhibit the TNDR effect. ***In the revised manuscript we attribute this difference to a larger concentration of oxygen vacancies in ALD-grown*** $\boldsymbol{HfO_2}$ as compared to the same defects in crystalline $SrTiO_3$ or mobile $K^+$ contaminants in $SiO_2$ (coming from the use of PTAS salt during direct CVD growth of $MoS_2$).

**Comment 6:**

*Clarification is needed regarding the reproducibility of the NDR behavior over repeated sweeps. It is important to distinguish whether this effect represents a stable functional mechanism or the onset of progressive dielectric degradation. Overall, the study is interesting, but addressing the points above would significantly strengthen the physical interpretation and practical implications of the work.*

**Answer:** We agree with the reviewer. As noted in our reply to your comment 1, ***we have added new Fig.S9 demonstrating reasonable cycle-to-cycle reproducibility of TNDR over 33 cycles***. The TNDR overshoot and window remain stable, confirming that the effect is a reversible ion drift rather than a progressive oxide degradation.

## Reviewer 3

**<u>Comment 0:</u>**

*Dear authors, The manuscript titled "Mobile charges in molybdenum disulfide/high-k oxide transistors: from abnormal instabilities to memory-like dynamics" presents a detailed characterization of the hysteretic behavior in MoS2/HfO2 and MoS2/Al203 field-effect transistors. The authors successfully highlight the transition from CW hysteresis at room temperature to CCW hysteresis with NDR at elevated temperatures. They propose a compact model attributing these effects to the drift of mobile oxygen vacancies within the oxide. While the physical mechanisms behind the hysteresis are discussed in depth, the memory-like dynamics mentioned in the title are not sufficiently explored and convincingly demonstrated. The current focus remains heavily on the physical explanation of high-temperature counterclockwise hysteresis rather than on practical memory applications or performance metrics. Therefore, while the work is technically interesting, it requires major revisions to better align the results with the stated claims before it can be considered for publication in Communications Engineering.*

**<u>Answer:</u>** We thank the reviewer for positive evaluation of our manuscript and agree that the title could better reflect the focus of this work on the CCW hysteresis and transient NDR (TNDR) analysis rather than practical memory applications. Thus we have now changed the title as "**Mobile charges in $MoS_2$/high-k oxide transistors: from abnormal instabilities to transient negative differential resistance**". Furthermore, in the revised version we have introduced the TNDR overshoot and TNDR window as the key metrics of the observed TNDR behavior. The detailed point-by-point replies are provided below.

**<u>Comment 1:</u>**

***MoS2 hysteresis is frequently associated with ambient adsorbates. While this study was conducted in vacuum, this detail is missing from the abstract and the specific vacuum level is not reported in the results, The authors should clarify these experimental conditions to ensure data reproducibility***

**Answer:** We agree with the reviewer. **However, the** vacuum level (base pressure ~$5\times10^{-6}$ torr) was mentioned in the Methods section of the previous version of the manuscript. ***Now we also mentioned it in page 4 of the manuscript (subsection "Investigated devices and measurement technique") to ensure better readability and said "in vacuum up to 275°C" in the abstract.*** Obviously, under these vacuum conditions and temperatures up to 275°C any contribution of ambient adsorbates is unlikely.

**Comment 2:**

*The authors claim the MoS2 under study is a monolayer, yet no AFM profile, flake image, or material characterization is provided to support this. Additionally, the optical image in Figure 1(b) should be shown at a higher magnification to clearly resolve the flake.*

**Answer:** We agree with the reviewer. ***In the new Fig.1b we now show Raman results which confirm the monolayer thickness of our commercial $MoS_2$ films, and the new Fig.1c (old Fig.1b) now uses a higher magnification optical image that clearly resolves the CVD-grown $MoS_2$ films.***

**Comment 3:**

*The output curves in the Figure 1(c) inset indicate Schottky behavior. While the compact model assumes a Schottky barrier of 0.2 eV, the manuscript does not specify if this value was derived from standard Richardson analysis or band-alignment observations. Since nonhomogeneous Schottky barriers can significantly enhance hysteresis in TMD-based FETs (see https://doi.org/10.1002/aelm.202500327), the authors should account for this effect in their discussion.*

**Answer:** We thank the reviewer for this remark. Actually in the equation (8) in the SI it is more correct to operate with the effective work function difference $\Phi_{MS}$ between the gate metal and the $MoS_2$ channel rather than with the Schottky barrier height. Then $\Phi_{MS}$ can be treated as the tuning parameter that lumps together the gate work function, fixed charges, Fermi level pinning, etc.

***We have adjusted this in the SI (page 8), also stating that $\Phi_{MS}$ = -0.2 eV was selected to position the subthreshold region similarly to the experimental $I_D$-$V_G$ curves (page 9 in the SI). This approximation when calculating $V_{FB}$ is acceptable in a qualitative model that targets to reproduce trends.*** We also note that non-homogeneous Schottky barriers are not expected to dominate under our measurement conditions, as the observed hysteresis shows strong temperature and sweep-rate dependence that is consistent with charge trapping at room temperature and ion drift at elevated temperatures. We have read the recommended reference and acknowledge that barrier inhomogeneities can affect device behavior, but our vacuum environment and symmetric contact design should minimize such effects.

**Comment 4:**

*The curves in Figure 2 are presented in a confusing manner. Specifically, the dashed line representing the other branch of the transfer curve is not defined in the text or the caption. Additionally, the band-alignment diagram in Figure 2(e) lacks labels for the energy levels, such as Ec and Ev. The authors should clarify these graphical elements to improve the readability of the data.*

**Answer:** ***We have revised Figs. 2-6 and added notes that solid lines correspond to the forward and dashed lines to the reverse sweep directions, in addition to the corresponding solid/dashed arrows showing different sweep directions. The band diagram in Fig. 2e now has clear labels for $E_C$ and $E_V$.***

**Comment 5:**

*The sentence "Furthermore, ΔVH starts increasing at smaller sweep frequencies as these ..." at page 8 lacks literature reference.*

**Answer:** We agree with the reviewer. ***As suggested, we have added literature references for this sentence on page 8 regarding the relationship between $\Delta V_H$, sweep frequency and dielectric trap capture/emission time constants*** (T. Knobloch et al., IEEE IEDM 2023 and Y.Z. Lv npj 2D Mater. Appl. 10 2026).

**Comment 6:**

*The increase in the ON current observed with increasing sweep times should also be considered in relation to potential temperature increments resulting from prolonged measurement durations. The authors should discuss whether self-heating contributes to this behavior.*

**Answer:** We thank the reviewer for this comment. Indeed, the observed increase in ON current with sweep time could in principle be contributed by self-heating. However, the effect is only present at 175°C and exclusively in $HfO_2$ devices, being absent at room temperature and in $Al_2O_3$ devices under identical bias. Furthermore, for our hysteresis sweeps we use small $V_D$=0.1V and the typically measured $I_D$ values are tens of μA. This suggests that the maximum power should be within several μW. Thus, self-heating would be negligible and, if present, cannot explain considerable increase in the ON current which we observe. We therefore conclude that the sweep-time dependence arises from thermally assisted oxygen-vacancy drift in $HfO_2$ rather than from Joule heating. ***A sentence about negligible contribution of Joule heating is now added in the end of page 8 in the manuscript.***

**Comment 7:**

*The authors explain that the CCW hysteresis and the NDR effect is also present in FETs at 125°C and with slower time of sweep and relate this possibility to devices with more vacancies in the oxide. This possibility makes more difficult the reproducibility and the control on the behavior of the device. The authors should discuss some engineering of defects method to clarify in which sense this type of device should be considered for memory-like dynamics.*

**Answer:** We thank the reviewer for this comment. We agree that device-to-device variability in oxygen vacancy concentration can affect the onset temperature of CCW/NDR behavior. However, this variability also offers a pathway for engineering: by controlling ALD conditions (e.g. deposition temperature, precursor pulse ratio or post-deposition annealing), the vacancy concentration can be intentionally tuned. This is analogous to controllable doping in conventional semiconductors. Such tunability is desirable for memory-like applications, as it allows the device response to be adjusted for

different operating windows (e.g. lower vacancy concentration shifts CCW/NDR to higher temperatures or slower sweeps). While a systematic defect engineering study is beyond the present scope, our results demonstrate that the mechanism itself is robust and reproducible across devices when measured under the same conditions. ***We have added a sentence about varying the density of oxygen vacancies during the ALD growth to the page 10 in the revised manuscript.***

**Comment 8:**

*Several parameters in the compact model appear arbitrary. The effective mobility of 10 cm2 V-1 s-1 for MoS2 should be justified by specifying if it was extracted via transfer curves, the Y-function method, or output curves. The assumed bandgap of 2.53 eV is significantly higher than the widely reported value of approximately 1.8 ev (https://doi.org/10.3389/fchem.2021.700250). The subthreshold swing of 280 mV/decade should also be verified against experimental data. Notably, the temperature dependence of the subthreshold swing could provide an estimation of interface state density and its role in the observed hysteresis (see again https://doi.org/10.1002/aelm.202500327). Finally, the selection of the Nmob value must be clearly explained to establish the reliability of the model.*

**Answer:** We thank the reviewer for this careful examination. We emphasize that ***our compact model is qualitative and intended only to illustrate the physical origin of the CCW hysteresis and NDR (mobile charge drift), not to provide precise quantitative fits.*** Therefore, the parameters are chosen as representative values within physically reasonable ranges:

- Bandgap (2.53eV): The reviewer correctly notes that the bandgap of monolayer $MoS_2$ is often reported as ~1.8 eV. However, this value corresponds to the optical bandgap (the A-exciton absorption peak) and thus it is incorrect to use 1.8eV when speaking about FETs. In contrast, just like in our all our previous works (see e.g. Yu.Yu. Illarionov et al, Nat. Commun. 2020) we use the electronic (transport) bandgap consistent with an established reference *(F. Rasmussen et al Phys. Chem. C. 119, 13169, 2015*). The difference between electronic and optical band gaps is given by the large exciton binding energy (~0.87 eV) in monolayer $MoS_2$ which arises from strong Coulomb interactions. ***We have now mentioned that it is electronic bandgap, see page 9 in the SI***

- Mobility (10 cm²/V·s) and SS (280 mV/dec): These are approximate values used only to produce realistic $I_D$–$V_G$ shapes; they do not affect the simulated ion drift dynamics

- $N_{mob}$: This is parameter only scales the magnitude of the CCW hysteresis and TNDR. Its absolute value is not unique, and the model is not calibrated to a specific device as there is no practical reason for this. Instead, ***in Fig.S4 we provide the modeling results for different*** $N_{mob}$ ***that is the most important to illustrate that the presence of TNDR requires sufficient*** $N_{mob}$***, while simple CCW hysteresis may be caused by smaller*** $N_{mob}$

- Interface states: Our sweep times range from over 10s to >10ks. Fast interface states do not contribute hysteresis in this time ranges (see e.g. https://ieeexplore.ieee.org/abstract/document/8347071 ). Hence, border oxide traps and mobile charges are the dominant sources of hysteresis

Given the qualitative nature of the model, we believe the current parameter selection is sufficient. A detailed parameter extraction (e.g., Y-function method) is beyond the scope of this mechanistic study.

**Comment 9:**

*The device stability under bias stress is well presented; however, the memory effect, despite being a central theme of the title, remains unclear. Essential metrics for a functional memory device, such as write/read/erase operations, endurance, and retention, are not reported. Consequently, the work currently functions as a characterization of hysteresis rather than a demonstration of a memory device. The authors should discuss these limitations and consider revising the title to better align with the data actually presented in the manuscript.*

**Answer:** We agree with the reviewer. The title is now adjusted as "**Mobile charges in $MoS_2$/high-k oxide transistors: from abnormal instabilities to transient negative differential resistance**". The abstract and conclusions now focus on TNDR rather than full memory functionality, and ***in the last sentence of Conclusions we now explicitly state that practical memory applications would require further engineering.***

## Reviewer 4

**Comment:**

*I co-reviewed this manuscript with one of the reviewers who provided the listed reports. This is part of the Communications Engineering initiative to facilitate training in peer review and to provide appropriate recognition for Early Career Researchers who co-review manuscripts.*

**Answer:** We thank the reviewer for the co-reviewing effort. All points raised by the primary reviewer have been addressed as detailed above.
